# Click Chemistry in Natural Product Modification

**DOI:** 10.3389/fchem.2021.774977

**Published:** 2021-11-17

**Authors:** Xiang Zhang, Shuning Zhang, Songfeng Zhao, Xuan Wang, Bo Liu, Hongtao Xu

**Affiliations:** ^1^ Department of Pharmacy, The First Affiliated Hospital of Zhengzhou University, Zhengzhou University, Zhengzhou, China; ^2^ Shanghai Institute for Advanced Immunochemical Studies, ShanghaiTech University, Shanghai, China; ^3^ The Second Clinical Medical College, Guangdong Provincial Key Laboratory of Clinical Research on Traditional Chinese Medicine Syndrome, Guangzhou University of Chinese Medicine, Guangzhou, China

**Keywords:** natural product, click chemistry, drug discovery, modular synthesis, triazole, sulfur(VI) fluoride exchange, pharmacological activity

## Abstract

Click chemistry is perhaps the most powerful synthetic toolbox that can efficiently access the molecular diversity and unique functions of complex natural products up to now. It enables the ready synthesis of diverse sets of natural product derivatives either for the optimization of their drawbacks or for the construction of natural product-like drug screening libraries. This paper showcases the state-of-the-art development of click chemistry in natural product modification and summarizes the pharmacological activities of the active derivatives as well as the mechanism of action. The aim of this paper is to gain a deep understanding of the fruitful achievements and to provide perspectives, trends, and directions regarding further research in natural product medicinal chemistry.

## Introduction

Natural products (NPs) are secondary metabolites that are produced by the evolutionary optimization of nature. They usually possess diverse and complex architectures and are endowed with versatile pharmacological activities, offering an abundant source for therapeutic drug discovery (Hunter, 2008; Jiménez, 2018) Newman and Cragg, 2020; Rodrigues et al., 2016; Li and Vederas, 2011). Natural product-based drug discovery can date back to the isolation of morphine, the first pharmacologically active pure natural product which was purified by Friedrich Sertürner more than 200 years ago. From then on, considerable works have been devoted to the synthesis of natural derivatives (Wang M. et al., 2018; Foley et al., 2020), and/or natural product-like screening libraries with the aim of therapeutic drug discovery (Huigens et al., 2013; Crane and Gademann, 2016; Ma et al., 2019; Wilson et al., 2020; Xie et al., 2020). These efforts have led to the discovery of various important clinical drugs, such as anticancer agents (e.g., taxol and doxorubicin), immunosuppressants (e.g., cyclosporine and doxorubicin), antimalarial agents (e.g., quinine and artemisinin) and lipid regulate drugs (e.g., lovastatin and relatives). Even today, natural products still serve as a fundamental source of diverse biological functions, facilitating the development of chemical biology and drug discovery.

**FIGURE 1:**
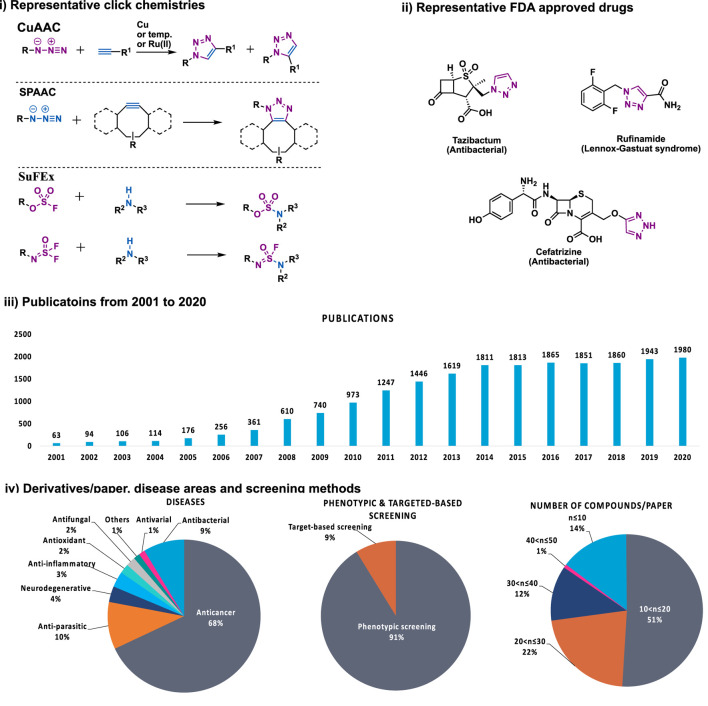
i) Representative click chemistries; ii) representative drugs synthesized by CuAAC; iii) number of papers published between 2001 and 2020 that contain the keywords click chemistry, SPAAC, IED-DA, sulfur fluoride exchange or 1,2,3-triazole according to Scopus; iv) number of compounds per paper, screening methods, disease areas of 137 papers that analyzed in this review.

As natural products are usually complex molecules with various stereo centers, sp3 carbon, and labile functionalities, the *de novo* synthesis of natural products or their derivatives always need complicated synthetic strategies, and accomplished in time-consuming multistep syntheses with low quantity and a limited number of derivatives. Therefore, chemistries that can be used for the late-stage functionalization and diversification of natural products are highly desirable, and should meet the following criteria: 1) reliable, selective, orthogonality to other functionalities; 2) modular, broad substrate scope; 3) high yield; 4) operational simplicity. In 2001, these criteria were codified by Professor K. Barry Sharpless, who termed such ideal chemistries as “click chemistry” (Kolb et al., 2001). From then on, click chemistry reactions, especially the Cu(I)-catalyzed Huisgen 1,3-dipolar cycloaddition between alkynes and azides (CuAAC) was quickly recognized as versatile players in the modification of various molecules, especially complex natural products, providing enhanced properties or new functions for chemical biology and drug discovery (Figure 1).

Despite the success of CuAAC, the search for other click chemistries has never stopped. Today several elegant click chemistries have been well developed, such as strain-promoted azide-alkyne cycloaddition (SPAAC), inverse electron-demanded Diels-Alder (IEED-DA), and Sulfur (VI) Fluoride Exchange (SuFEx) chemistry. These chemistries have played a key in chemical biology and drug discovery, particularly the emerging SuFEx chemistry, another ideal click reaction proposed by Professor Sharpless in 2014 (Dong et al., 2014; Barrow et al., 2019), have already gained wide application in the synthesis of drug screening libraries (Kitamura et al., 2020; Smedley et al., 2020), late-stage modification of drugs and natural products (Li S. et al., 2017; Liu et al., 2018), DNA-encoded library synthesis (Liu et al., 2019; Xu H. et al., 2019; Zhang et al., 2021), and the synthesis of ^18^F radio tracers (Zheng et al., 2021).

Indeed, now that natural products have met with click chemistry, a new era of natural product-based drug discovery has come. Previously, several elegant review papers have summarized the CuAAC reaction in medicinal chemistry, mainly focused on the synthesis of 1,2,3-trizaoles for various properties such as anti-cancer (Xu Z. et al., 2019; Liang et al., 2021), anti-bacterial, etc., (Kacprzak et al., 2016; Rani et al., 2020; Guo et al., 2021; Kumar et al., 2021; Serafini et al., 2021). Especially, this review focuses on the late-stage modification of natural products by using not only the CuAAC reaction but also other click chemistries such as SPAAC and especially the emerging SuFEx chemistry (Table 1) (Dong et al., 2014), and thus encompasses a much wider variety of natural product derivatives and the corresponding pharmacological activities. These natural product derivatives are classified according to their structural features, covering a time span mainly of the last decade.

**TABLE 1:** Typical reaction conditions of click chemistry in this paper.

Click chemistries	Conditions	Reference
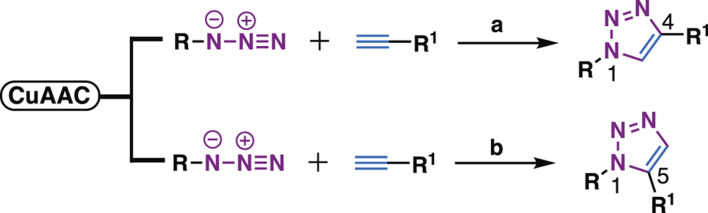	a) azide (1 equiv), alkyne (1–2 equiv), sodium ascorbate (10 mol%), CuSO_4_·5H_2_O (5% mol), *t*-BuOH–H_2_O, room temperature	Bahia et al. (2016); Thomopoulou et al. (2016); Li et al. (2018)
	b) azide (1 equiv), alkyne (1–2 equiv), Cp*RuCl(PPh_3_)_2_ (10 mol%), dichloromethane, 50°C	
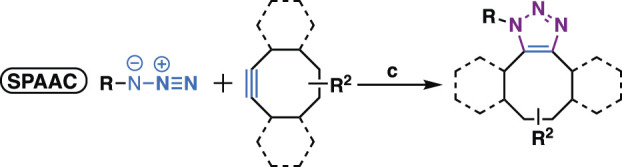	c) azide (1 equiv), DBCO (1 equiv), DMSO, room temperature	Sinha et al. (2016)
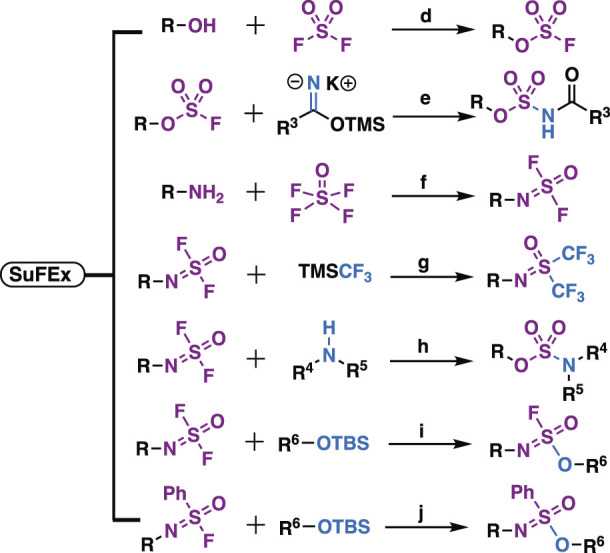	d) phenol (1 equiv), SO_2_F_2_ (balloon), Et_3_N or DIPEA (2 equiv), dichloromethane or DMF, room temperature	Li et al. (2017a); Gao et al. (2018); Smedley et al. (2019); Smedley et al. (2020)
	e) arylfluorsulfate (1 equiv), trimethylsilyloxyl imidate (1.2 equiv), anhydrous DMF, room temperature	
	f) amine (1 equiv), SOF_4_ (balloon), Et_3_N or DIPEA (2.15 equiv), CH_3_CN, room temperature	
	g) iminosulfur oxydifluoride (1 equiv), TMSCF_3_ (2.2 equiv), KFHF (1 mol%), DMSO, room temperature	
	h) iminosulfur oxydifluoride (1 equiv), amine (2 equiv), CH_3_CN, room temperature	
	i) iminosulfur oxydifluoride (1 equiv), ArOTBS (1 equiv), DBU (10 mol%), CH_3_CN, room temperature	
	j) sulfonimidoyl fluoride (1 equiv), ArOTBS (1 equiv), DBU (30 mol%), CH_3_CN, 60°C	

The aim of this paper is to showcase the state-of-the-art development of click chemistry in natural product modification, thereby gain a deep understanding of the fruitful achievements, and provide perspectives, trends, and directions regarding further research in natural product medicinal chemistry.

## Click Chemistry-Based Modification of Terpenoids

Terpenoids, also known as isoprenoids, are the largest class of plant secondary metabolites, representing 60% of the known natural products. Terpenoids derive from 5-carbon isoprene and usually have oxygen-containing functionalities. Many diterpenoids, especially cyclic sesqui-, di- and triterpenoids are endowed with bewildering structural features such as multiple chiral centers, rigid skeleton, diverse functionalities, and the consequent various promising biological activities. Moreover, some of the terpenoids are readily available from nature, thereby can serve as inexhaustible starting materials for the synthesis of bioactive natural products or natural product-like drug screening libraries (Xu et al., 2014b). By arching azide or alkyne at the suitable position of terpenoids, followed by reactions with various alkyne or azide containing building blocks, a lot of terpenoid derivatives have been synthesized and evaluated for various biological activities.

**FIGURE 2:**
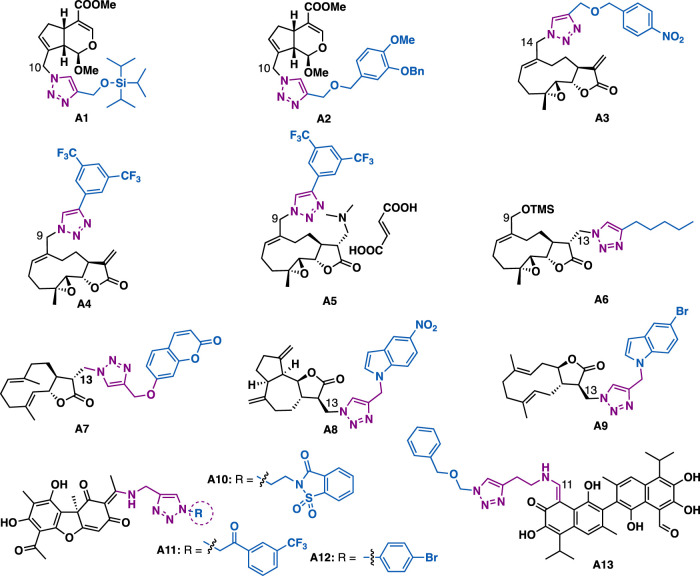
Representative iridoid-, sesquiterpenoid-, and bisesquiterpenoid-triazole derivatives.

Genipin (Figure 2), an iridoid derived from *Gardenia jasminoides* Ellis, has been reported to possess various biological activities such as anticancer and antioxidant. The introduction of triazole moiety into genipin has been demonstrated to generate derivatives with improved cytotoxicity. For example, C10 genipin-triazole **a1** (P-388: IC_50_ = 2.54 *μ*M; A549: IC_50_ = 4.53 *μ*M) and **a2** (P-388: IC_50_ = 5.49 *μ*M; A549, IC_50_ = 4.81 *μ*M) could exert more potent inhibitory activity than the parental genipin against tumor cells (P-388: IC_50_ = 11.12 *μ*M; A549 > 20 *μ*M) (Silalai et al., 2020). Molecular docking studies have indicated that a2 engaged four hydrogen bonds in the colchicine binding site of tubulin (Asn-*β*-258 and the carbonyl group of genipin (Silalai et al., 2020), Asn-*β*-249 and oxygen atom of ether moiety of triazole, Ala-*β*-250 and the triazole, and Glu-α-183 and methoxy group of benzylether).

Melampomagnolide B, a sesquiterpene lactone identified from *Magnolia grandiflora*, could exert moderate inhibitory activity against Bel7402, HCT-116, PANC1, A549, and U87 cancer cell lines (IC_50_ = 4.93–10.86 *μ*M). Whereas, C13 triazole containing derivative a3 could exert improved inhibitory activity (IC_50_ = 0.43–1.54 *μ*M), (Ding et al., 2018), and especially it could exert submicromolar inhibitory activity against HCT116 cells with IC_50_ value of 0.43 *μ*M, which is 11.5 times more potent than melampomagnolide B (IC_50_ = 4.93 *μ*M). Preliminary mechanistic studies indicated that a3 could induce apoptosis and inhibit the proliferation and migration of the tested HCT-116 cells. Crooks et al. reported that melampomagnolide B-triazole a4 could exert low micromolar to submicromolar (GI_50_ = 0.02–1.86 *μ*M) inhibitory activity against the growth of a panel of 60 cancer cell lines derived from different organs including lung, CNS, leukemia, colon, renal, melanoma, ovary, prostate and breast cancer cells (Janganati et al., 2018), and especially it could exert nanomolar (EC_50_ = 0.4–0.7 *μ*m) inhibitory activity against two clinical AML specimens. In addition, salt a5, a water-soluble prodrug of a4, could also exert comparable inhibitory activity as a4 in primary AML cells. Whereas, the C13 melampomagnolide B-triazole derivatives generally showed reduced inhibitory activity, with only a6 (GI_50_: 46 – 76 *μ*M) showed marginal inhibitory activity against the tested cancer cell lines (A549, MCF-7, SKMEL-28, and Hs683), implying the *a*-methylene-*c*-lactone motif may play a key role in retaining the potent cytotoxicity (Zaki et al., 2016).

Babu et al. synthesized 20 triazole containing derivatives by Michael addition of the *α*-methylene-*c*-lactone motif of costunolide and dehydrocostus by sodium azide, followed by reaction with various terminal alkynes under CuAAC. *In vitro* data indicate that costunolide-triazole-coumarin a7 (IC_50_ = 0.12 *μ*M) could exert more potent cytotoxic activity than the precursor costunolide (IC_50_ = 0.56 *μ*M) in MDA-MB-231 cells (Pavan Kumar et al., 2016). Similarly, indole-triazole-dehydrocostus-lactone a8 (IC_50_ = 0.16 *μ*M) could exert more potent cytotoxic activity than dehydrocostus-lactone (IC_50_ = 0.56 *μ*M) in MDA-MB-231 cells, while derivative a9 (IC_50_ = 0.68 *μ*M) showed more potent cytotoxic activity than dehydrocostus-lactone (IC_50_ = 4.1 *μ*M) in IMR-32 cells (Pavan Kumar et al., 2016).

Usnic acid is a dibenzofuran secondary metabolite that is isolated from lichen genera. Usnic acid-triazole-saccharin hybrid a10 (MIC = 2.5 *μ*M) could exert slightly better inhibitory activity than clinical drug isoniazid (MIC = 2.9 *μ*M) against *Mycobacterium tuberculosis* (Mtb) (Bangalore et al., 2020), but failed to show any antibacterial activity against *Bacillus subtilis*, while hybrid a11 (MIC = 40.9 *μ*M) could exert good antibacterial activity against *Bacillus subtilis*. Molecular docking studies indicated that the usnic acid moiety of a10 could occupy the active site of Mtb enzyme enoyl reductase (InhA), while the oxygen of sulfamide in saccharin could engage a hydrogen bond with GLN100, and the triazole moiety could have π−π stacking interaction with PHE97. Guo et al. reported that usnic acid-triazole hybrid a12 could exert selective anti-*Toxoplasma gondii* activity with a good selectivity index (IC_50_ = 261 *μ*M, SI = 1.34), which is slightly better than the reference drugs sulfadiazine (SI = 1.15), pyrimethamine (SI = 0.89), and spiramycin (SI = 0.72) and also the parental (+)-usnic acid (SI = 0.96) (Guo et al., 2019).

Gossypol is a natural yellow pigment bi-sesquiterpene that acts as a plant defense system against insects and fungi. Pyta et al. reported that gossypol-triazole a13 (MIC = 16 *μg*/ml) could exert comparable inhibitory as miconazole against *Fusarium oxysporum* (MIC = 16 *μ*g/ml). Mechanistic studies indicated that a13 might inhibit biosynthesis of ergosterol, thereby exerting its anti-fungal activity (Pyta et al., 2016).

**FIGURE 3:**
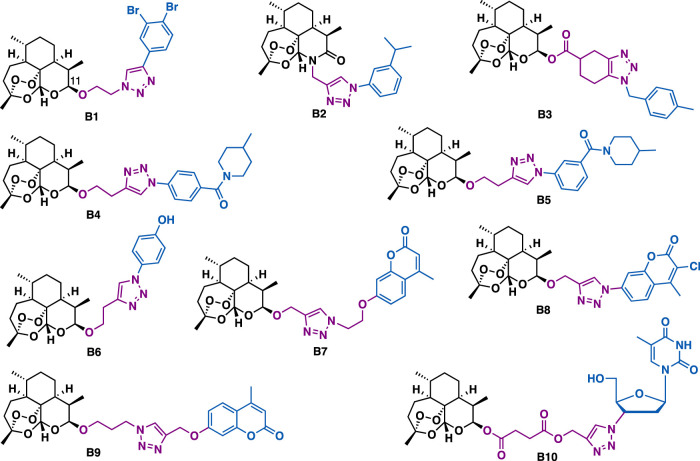
Representative artemisinin derivatives.

Artemisinin derivatives with peroxide-containing sesquiterpenoid lactone structures such as artesunate, arteether, and dihydroartemisinin are the main chemotherapeutic drugs for malaria. Beyond their intrinsic antimalarial potential, artemisinin derivatives, particularly derivatives that are conjugated with another pharmacophore by triazole could exert promising anticancer activity. For example, Dehaen et al. reported that C11 artemisinin-triazole b1 (CEM: IC_50_ = 0.92 *μ*M; Hela: IC_50_ = 1.2 *μ*M) could exert more potent inhibitory activity than b2 (CEM: IC_50_ = 2.7 *μ*M; Hela: IC_50_ = 11 *μ*M) and b3 (CEM: IC_50_ = 10 *μ*M; Hela: IC_50_ = 16 *μ*M) (Figure 3), indicating the linker between triazole and artemisinin could influence their potential (Jana et al., 2017).

Binh et al. reported that artemisinin-triazole b4 (IC_50_: 2.5–4.7 *μ*M) could exert more potent inhibitory activity than its regio-isomer b5 (IC_50_: 13.5–19.9 *μ*M) and dihydro artemisinin (DHA, IC_50_: 39.9–84.3 *μ*M) against a panel of cancer cell lines including P388, MCF-7, HL-60, and LU-1 cells, indicating the position of the amide substituent on the benzene was critical to the potent inhibitory activity (Binh et al., 2016). Bhakuni et al. reported that b6 could inhibit the growth of a panel of cancer cell lines (IC_50_ = 4.06–36.65 *μ*M) by arresting cell cycle (G2/M phase) and inducing apoptosis in lung and skin cancer cells (Kapkoti et al., 2018).

Hybridization of dihydroartemisinin with other natural products or drug molecules is another good strategy to obtain potential anticancer compounds. Artemisinin-coumarin hybrid b7, b8, and b9 could only exert moderate cytotoxic activity against MDA-MB-231, HCT-116, and HT-29 cancer cells under normoxic conditions (Tian et al., 2016; Yu et al., 2018). Whereas, under anoxic conditions, hybrid b7 (anoxic, IC_50_ = 0.05 *μ*M; normoxic, IC_50_ = 17.7 *μ*M) and b8 (anoxic, IC_50_ = 0.01 *μ*M; normoxic, IC_50_ = 1.5 *μ*M) showed 334-fold and 150-fold more potent than that under normoxic conditions in HT-29 cells, which is probably associated with the high expression of CA IX on the membrane of HT-29 cells. While, hybrid b9 showed a 41.38-fold and 20.03-fold higher activity than that under normoxic conditions in HCT-166 (anoxic, IC_50_ = 0.43 *μ*M; normoxic, IC_50_ = 17.96 *μ*M) and MDA-MB-231 cells (anoxic, IC_50_ = 3.62 *μ*M; normoxic, IC_50_ = 72.5 *μ*M), respectively. Structure activity relationship (SAR) studies indicated that the spacer between triazole-artiartemisinin and triazole-coumarin as well as the substituents on the coumarin were critical to the selective inhibitory activity. Artemisinin-azidothymidine hybrid b10 (IC_50_ = 16.5 *μ*M) could exert more potent antiproliferative activity than artesunate (IC_50_ = 78.5 *μ*M) against KB cancer cells (Tien et al., 2016), indicating the azidothymidine moiety plays a key role in the increased activity. In addition, SAR studies showed that hybrids with ester triazole-linker could exert more potent antiproliferative activity than hybrids with amide triazole-linker.

**FIGURE 4:**
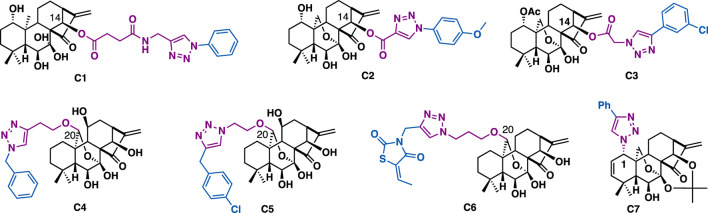
Representatives oridonin derivatives.

Oridonin is an *ent-*kaurene diterpenoid that was initially isolated from various *Isodon* species, which was widely used as home remedy herb medicine in China and Japan. Oridonin-triazoles generally showed broad-spectrum anticancer activity. For example, C14 oridonin-triazole C1 (HTC116: IC_50_ = 6.89 *μ*M; MCF7: IC_50_ = 6.81 *μ*M), C2 (HTC116: IC_50_ = 1.94 *μ*M; MCF7: IC_50_ = 3.83 *μ*M) (Figure 4) (Shen et al., 2019), C3 (PC-3: IC_50_ = 3.1 *μ*M; LNCaP: IC_50_ = 4.1 *μ*M) could exert more potent anti-proliferative activities than that of oridonin (IC_50_ = 16.28–24.80 *μ*M) (Hou et al., 2019a). In addition, they could effectively overcome drug resistance and showed weak cytotoxicity on non-cancer cells. SAR studies indicated that the phenyl 1,2,3-triazole moiety and the linker between oridonin and triazole play a key role in improving antiproliferative activity. Preliminary mechanistic studies indicated that C3 could arrest cell cycle (G2/M phase) and induce apoptosis of PC-3 cells. Through the introduction of azide or alkyne linkers at the C20 hydroxyl group, Liu et al. synthesized a focused library of Jiyuan oridonin A-triazoles, *in vitro* data indicated that all the triazole derivatives could exert good anti-proliferative activities. Among them, C4 (IC_50_ = 4.26–8.95 *μ*M) and **C5** (IC_50_ = 2.70–5.04 *μ*M) could exert broad-spectrum inhibitory activity against a panel of cancer cell lines including Eca109, EC9706, SMMC7721, and MCF7. Mechanistic studies indicated that C5 could promote intracellular ROS level, arrest cell cycle (G2/M phase) and significantly induce cell apoptosis in the tested MGC-803 cells (Ke et al., 2018b). Later, the same group reported that C6 (IC_50_ = 0.6–5.0 *μ*M) could exert potent anti-proliferative activities on several cancer cell lines (Eca109, EC9706, SMMC7721, and MCF7) with good selectivity towards normal cells. Mechanistic studies indicated that C6 could inhibit cell migration with the Wnt signaling pathway involved, arrest cell cycle (G1 phase), and induce cell apoptosis in the tested SMMC-7721 cells (Ke et al., 2018a). Due to the challenge of the introduction of azide at C1 of oridonin, there are only limited C1 triazole derivatives have been reported, and according to the reported data, derivative C7 could exert submicromolar inhibitory activity on the tested cancer cells (MCF-7: IC_50_ = 0.38 *μ*M; MDA-MB-231: IC_50_ = 0.48 *μ*M) (Ding et al., 2013), implying the introduction of triazole at C1 of oridonin was tolerated.

**FIGURE 5:**
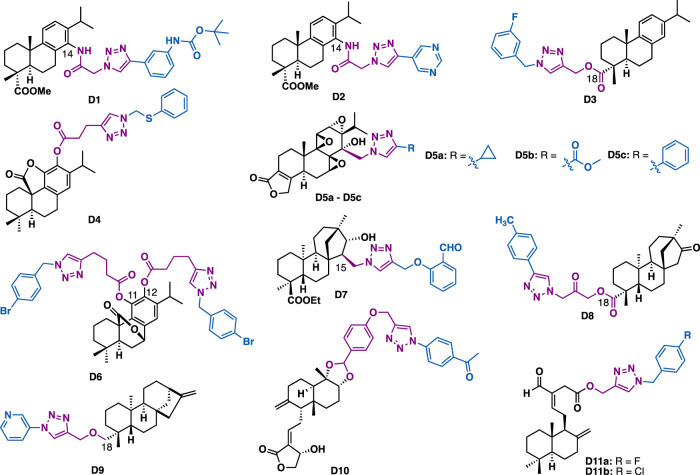
Representative abietane, *ent*-beyerane, kaurene and labdane diterpene derivatives.

Abietane-type diterpenes are a series of tricyclic terpenoids that possess various biological activities. For example, dehydroabietic acid and abietic acid are readily available diterpenoids that can be isolated from disproportionated rosin. They have been widely used as starting materials for the synthesis of natural products or natural product-like drug screening libraries (Xu et al., 2014b; Xu et al., 2017). With the installation of azide or alkyne functionalities at C14 or C18, several series of dehydroabietic acid-triazole derivatives were synthesized. The screening of antiproliferative and antibacterial activities indicated that D1 could inhibit the growth of several cancer cell lines (IC_50_ = 0.7–1.2 *μ*M) (Figure 5) (Hou et al., 2017a). Mechanistic studies indicated that D1 could induce apoptosis of MDA-MB-231 cells. In addition, the C18 triazole derivative D3 (IC_50_ = 5.90 *μ*M) could exert comparable inhibitory activity to the reference drug cisplatin against HepG2 cells (IC_50_ = 6.42 *μ*M) (Li et al., 2019), and derivative D2 could exert antibacterial activities against both Gram-positive bacteria (*Bacillus subtilis* and *Staphylococcus aureus*) and Gram-negative bacteria (*Escherichia coli* and *Pseudomonas fluorescens*) strains (MIC = 1.6–3.1 mg/ml) with good drug-like properties and low cytotoxicity in noncancerous mammalian cells (Hou et al., 2017b).

Carnosic acid and carnosol are phenolic diterpenes that were identified from rosemary and mountain desert sage. Carnosic acid *γ*-lactone-triazole D4 could exert moderate inhibitory activity on MRC-5 (IC_50_ = 45.1 *μ*M) and AGS (IC_50_ = 39.2 *μ*M) cancer cells (Pertino et al., 2015), while C11, C12 carnosol-bistriazole D6 (MIC = 125 *μ*g/ml) could inhibit the growth of *C. neoformans* at the concentration of 250 *μ*g/ml (Pertino et al., 2015).

Triptolide is an abietane-type diterpene that was identified from *Tripterygium wilfordii Hook.*f (TWHF). It has the unique structural features of three successive epoxides and an unsaturated *α*, *β*-lactone. Due to its various promising biological activities, considerable work has been devoted to its total synthesis, structural modification, and targeted delivery with the aim to reduce its toxicity (Hou et al., 2019b; Xu and Liu, 2019; Zhang X. et al., 2019). With the introduction of azidomethyl at C14 of triptolide, Li et al. synthesized a series of C14 triazole substituted epi-triptolide derivatives D5a, D5b, and D5c (Xu et al., 2014a). *In vitro* data indicated that these derivatives only showed weak cytotoxic activity as compared to triptolide. However, considering various promising biological activities of triptolide (Hou et al., 2019b), further evaluation of their biological activates such as neuroprotective and anti-inflammatory activities are highly expected.

Isosteviol is a tetracyclic *ent*-beyerane diterpene that is endowed with multifarious bioactivities and can be readily isolated from *stevia* plant. Diversification of isosteviol by click chemistry at C19 or C15 could generate derivatives with potential anticancer activity. Tao et al. reported that C15 isosteviol-triazole D7 (IC_50_ = 2.987 *μ*M) could exert slightly better inhibitory activity than cisplatin (IC_50_ = 3.906 *μ*M) against HCT-116 cells (Liu et al., 2016). Quan et al. reported that C19 isosteviol-triazole D8 could inhibit the growth of several cancer cell lines (HCT-116, BEL-7402, and HepG2) with IC_50_ values in the range of 5.38–15.91 *μ*M, and that was 1.3- to 4.6-fold more potent than the reference drug 5-fluorouracil, and 6.3- to 16.8-fold more potent than the parental isosteviol (Luan et al., 2019). Mechanism studies indicated that D8 could inhibit colony formation and arrest cell cycle (S phase) in HCT-116 cells.

Kaurenoic acid (KA) is a kaurene diterpene that can be isolated from the fruits of *X. aethiopica*. Oliveira et al. reported that C19 kaurenoic acid-triazole D9 could exert moderate antimalarial activity (IC_50_ = 53 *μ*M) (De Santos et al., 2016), together with good selectivity (selective index (SI) = 774).

Both andrographolide and (*E*)-labda are labdane diterpenes. Andrographolide is one of the principal biological active compounds of *andrographis paniculate*, a traditional herb medicine widely used in China and India for the treatment of multiple diseases such as inflammation and cancer. Chinthala et al. reported that andrographolide triazole derivative D10 could exert selective inhibitory activity against K562 cancer cells with IC_50_ values of 8 *μ*M.(Chinthala et al., 2016). *In silico* docking studies indicated that D10 could bind with transient receptor potential vanilloid 1 (RPV1). (*E*)-labda is isolated from fresh rhizomes of *Curcuma amada*. Somappa et al. reported that labdane triazole derivatives D11a (IC_50_ = 0.75 *μ*M) and D11b (IC_50_ = 0.77 *μ*M) could exert excellent pancreatic lipase (PL) inhibitory activity, slightly better than the reference drug orlistat (IC_50_ = 0.8 *μ*M) (Jalaja et al., 2018).

Triterpenes represent an important class of natural terpenoids that are composed of three terpene units. They are endowed with the abilities to balance hormones, blood pressure, circulation, and digestion, and they also have been documented with anti-viral and anti-inflammatory activities.

**FIGURE 6:**
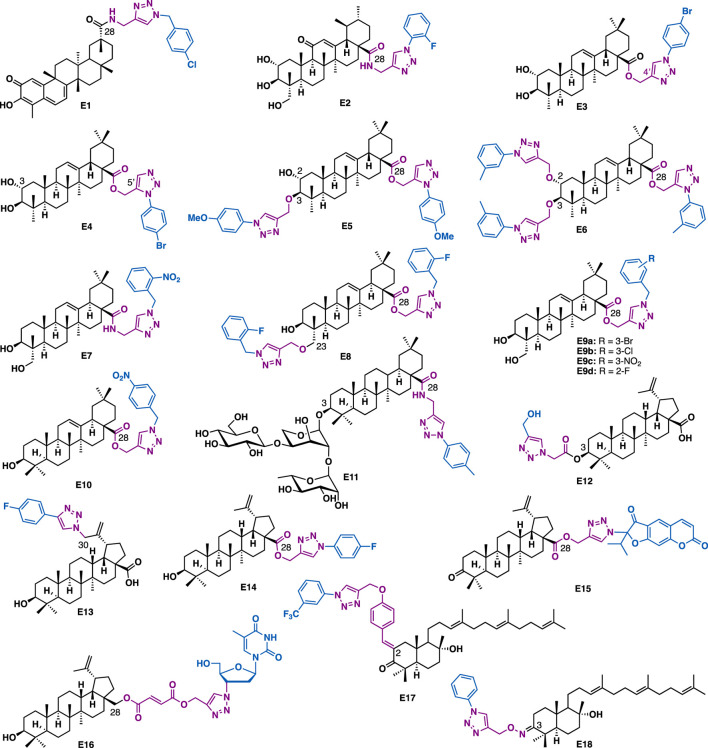
Representative triterpene derivatives.

Celastrol is a pentacyclic nortriterpene that is isolated from the root of *Tripterygium wilfordii* and/or *Triptergium regelii*. SAR studies indicated that its quinone methide moiety plays a key role in exerting various promising biological activities, thereby its structural modification was mainly conducted at C28 carboxyl group (Hou et al., 2020). For example, Zhang et al. reported that the C28 celastrol-triazole E1 could exert submicromolar anti-proliferative effect against AGS (IC_50_ = 0.97 *μ*M), HCT-116 (IC_50_ = 0.78 *μ*M) and BEL-7402 (IC_50_ = 0.63 *μ*M) cancer cell lines (Figure 6) (Zhang H.-J. et al., 2018).

Asiatic acid (AA) is a pentacyclic triterpenoid that was identified from the tropical herb medicine *Centella asiatica*. It has been reported to possess various biological activities such as antiinflammation, antidiabetics, and antitumor. Huang et al. reported that AA-triazole E2 could bind to NF-*κ*B (KD = 0.36 *μ*M) and exert low micromolar inhibitory activity against TNF-*α*-induced NF-*κ*B activation (IC_50_ = 0.14 *μ*M) (Huang et al., 2019). Molecular docking studies indicated that E2 could fit well in the active site of NF-*κ*B. The fluorine of benzene could form one hydrogen-bonding interaction with DNA chain (DA6), while the benzene could engage π−π interactions with PHE307 (Huang et al., 2019). Notably, 1,2,3-triazole as a hydrogen acceptor could establish four hydrogen bonds with amino hydrogen of LYS272 and DA5, indicating that the triazole moiety was crucial for the improved potency of E2. C-2 and C-3 hydroxy groups of AA could form two hydrogen bonds with the DNA backbone P=O of DG2. Also, C-23 hydroxy of AA formed two hydrogen bonds with LYS249. Moreover, the pentacyclic skeleton of AA moiety was surrounded by LYS241, PRO243, SER246, ASN247, LYS249, ASP271, and LYS272 via the hydrophobic interaction. Further, mechanistic studies indicated that E2 could inhibit NF-*κ*B DNA binding, nuclear translocation, and I*κ*B*α* phosphorylation. *In vitro* data showed that E2 could inhibit the growth of A549 cells (IC_50_ = 2.67 *μ*M) by at least partial inhibition of the activity of NF-*κ*B, as well as cell apoptosis and migration.

Maslinic acid is a pentacyclic triterpenoid that is isolated from pomace olive (*Olea europea L.*). It is reported that the introduction of triazole into maslinic acid could promote its anti-inflammatory activity (Chouaïb et al., 2016). For the mono triazole derivatives, the triazole 1’,4’-regioisomers have more potent anti-inflammatory activity than the 1’,5’-regioisomers, for example, E3 (IL-1*β* production = 21%; 100 *μ*M) is more potent than E4 (IL-1*β* production = 61%; 100 *μ*M). Regarding the bistriazole derivatives, bis-3,28-disubstituted triazoles were more potent than the bis-2,28-disubstituted triazoles, with derivative E5 showed the highest inhibitory activity (IL-1*β* production = 34%; 100 *μ*M). While 2,3,28-trisubstituted hybrids were the most potent series of all the triazole derivatives. Among them, E6 showed the highest potency (IL-1*β* production = 23%; 30 *μ*M). These data indicated that both the number and the position of the triazole were critical to the promising anti-inflammatory activity.

Hederagenin is an oleane-type pentacyclic triterpenoid that can be isolated in large quantities from *Sapindus saponaria* L*.* Hederagenin-triazole derivatives could not only exert anti-cancer activity but also have anti-leishmanial activity. For example, Barbosa et al. reported that E7 (IC_50_ = 2 *μ*M, SI = 22.5) could inhibit the growth of *Leishmania infantum* with a higher selectivity index as compared to the commercial drug potassium antimonyl tartrate trihydrate (IC_50_ = 80 *μ*M, SI = 0.1) (Rodríguez-Hernández et al., 2016a), highlighting its potential in anti-leishmanial drug development. Later, the same group reported E8, in which two triazoles were installed at C3 and C28, respectively, could also inhibit the growth of intracellular amastigote forms of *Leishmania infantum* with a good selectivity index (IC_50_ = 5.6 *μ*M, SI = 178) (Rodríguez-Hernández et al., 2017). C28 Hederagenin-triazole derivatives containing ester linkers are generally showed more potent cytotoxic activities than those that containing amide linkers (Rodríguez-Hernández et al., 2016b). *In vitro* antiproliferative activity data indicated that E9a (IC_50_ = 3.2–4.0 *μ*M), E9b (IC_50_ = 3.1–4.0 *μ*M), and E9c (IC_50_ = 3.2–4.1 *μ*M) could exert potent inhibitory activity against a panel of human cancer cell lines including 518A2, A2780, A549, HT-29, MCF-7, and 8505C, and are at least eight times more potent than the parental hederagenin, while E9d could exert selective inhibitory activity against HT-29 cells (IC_50_ = 1.6 *μ*M) with low toxicity in normal NIH 3T3 cells.

Oleanolic acid is a natural pentacyclic triterpenoid related to betulinic acid. It is widely distributed in many medicinal herbs in the form of free acid or saponin glycosides. Derivative E10, a C28 triazole derivative (Wei et al., 2015), in which the triazole moiety was installed by an ester linker, could exert potent antiproliferative activities against A375-S2 (IC_50_ = 4.97 *μ*M) and HT1080 (IC_50_ = 3.51 *μ*M) cancer cells. Meanwhile, its C3 saponin triazole relative, C28 hederacolchiside-triazole E11 (IC_50_ = 0.54–2.66 *μ*M) could exert more potent inhibitory activity than 5-fluorouracil (IC_50_ = 8.45–69.07 *μ*M) against a panel of cancer cell lines including PC3, HT29, HepG2, A549, HL60, and U937 (Li et al., 2018). Mechanistic studies indicated that E11 could arrest cell cycle (G1/S) and induce apoptosis of HepG2 cells.

Betulinic acid is a pentacyclic lupane-type triterpenoid that is isolated from the stem bark of *Platanus orientalis* and many other plants such as the birch trees *Ziziphus spp*., *Syzygium spp*., *Paeonia spp*., etc. So far, several series of C3, C28, and C30 betulinic acid-triazole derivatives have been synthesized and evaluated for various biological activities. Chakraborty et al. reported that C3 betulinic acid-triazolide E12 (IC_50_ = 14.9 *μ*M) could exert more potent inhibitory activity than betulinic acid and 5-fluorouracil in HT-29 cells (Chakraborty et al., 2015). Mechanistic studies indicated that E12 could induce apoptosis of cancer cells. Biophysical data showed that E12 could act as a DNA minor groove binder. Sangwan et al. reported that C28 betulinic acid triazole derivative E14 could exert promising inhibitory activity in a panel of cancer cell lines (HL-60: IC_50_ = 7 *μ*M; MiaPaCa2: IC_50_ = 5 *μ*M; PC-3: IC_50_ = 7 *μ*M; and A549: IC_50_ = 7 *μ*M) (Khan et al., 2016). Mechanistic studies indicated that E14 could block cell cycle at the G1 phase, induce mild cell apoptosis via both intrinsic and extrinsic pathways in HL-60 cells. Lipeeva et al. reported that C28 betulonic acid-furocoumarin oreoselone hybrid E15 could significantly reduce histamine-induced paw edema (edema index: 24.5%) to a level that was comparable to the nonsteroidal anti-inflammatory drug indomethacin (edema index: 22.4%) (Lipeeva et al., 2020). Shi et al. reported that C30 betulinic acid-triazole derivative E13 (IC_50_ = 1.3 *μ*M) could exert more potent antiproliferative than betulinic acid (IC_50_ = 11.5 *μ*M) in HL-60 cells (leukaemia) (Shi et al., 2015). Kiem et al. reported that C28 botulin-triazole-AZT hybrid E16 could exert promising antiproliferative activity against both KB (IC_50_ = 0.38 *μ*M) and HepG2 (IC_50_ = 1.32 *μ*M) cancer cells (Anh et al., 2017). Clearly, hybrid E16 showed much more potent inhibitory activity than AZT (IC_50_ > 400 *μ*M for both cell lines), implying the added value of merging betulin and AZT into a single hybrid.

Myrrhanone C is a natural bicyclic triterpene that is isolated from the gum resin of *Commiphora mukul*. By modification of C2 methene and C3 keto, Uppuluri et al. synthesized 27 myrrhanone C-triazoles (Poornima et al., 2015). *In vitro* data indicated that E17 could selectively inhibit the growth of DU-145 (IC_50_ = 13.8 *μ*M) and HepG2 (IC_50_ = 9.332 *μ*M) cancer cells, while E18 (IC_50_ = 6.16–9.59 *μ*M) could exert pan inhibitory activity against a panel of cancer cell lines (A549, Hela, MCF-7, DU-145, and HepG2). Both of them were more potent than the precursor myrrhanone C (IC_50_ = 12.02–26.61 *μ*M). Mechanistic studies indicated that E18 could arrest cell cycle (G2/M phase) and induce cell apoptosis.

## Click Chemistry-Based Modification of Alkaloids

Alkaloids are a series of nitrogen-containing compounds of plant origin, they usually possess various pronounced physiological effects on humans and other animals, such as morphine, quinine, strychnine, nicotine, and ephedrine.

**FIGURE 7:**
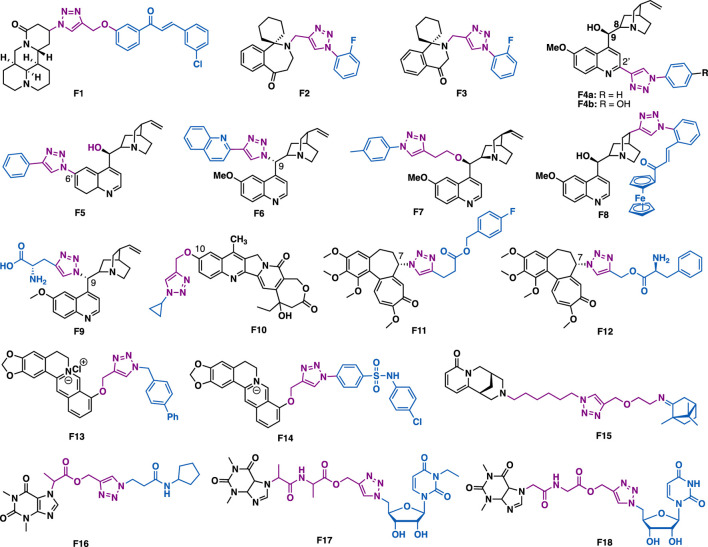
Representative alkaloid derivatives.

Matrine is a quinolizidine alkaloid that is isolated from the root of *Sophora flavescens* Ait (also known as Kushen), which is a traditional Chinese herb medicine that has been used for the treatment of liver diseases for thousands of years. Zhao et al. reported that matrine-triazol-chalcone hybrids could inhibit the growth of a panel of cancer cells (Figure 7) (Zhao et al., 2015). Among them, F1 (IC_50_ = 5.01–7.31 *μ*M) could exert broad-spectrum anticancer activities against a panel of cancer cell lines (A549, Bel-7402, Hela and MCF-7). Notably, F1 is more potent than the combination of matrine and chalcone (IC_50_ > 50 *μ*M), and also 5-fluotouracil (IC_50_ = 8.93–40.38 *μ*M). SAR studies indicated that the *α*, *β-*unsaturated ketone moiety and the triazole together might play a key in determining the promoted inhibitory activity. Further studies indicated that F1 could induce apoptosis in A549 cells, and suppress tumor growth in A549-xenografted nude mouse model (10 mg/kg) with no apparent cytotoxicity.

Homoharringtonine and homoerythrina are naturally occurring alkaloids that are isolated from *genus Cephalotaxus*. Their derivatives have been reported to exert various biological activities, and especially anticancer activity. Li et al. reported that homoerythrina-triazole F2 (IC_50_ = 1.89–4.19 *μ*M) could exert more potent inhibitory activity than rucaparib (IC_50_ = 4.91–13.51 *μ*M) and harringtonine (IC_50_: 10.55–11.71 *μ*M) in A549, HCT-116, and MCF-7 cancer cells (Li et al., 2020a). Mechanistic studies indicated that F2 could arrest cell cycle at the S phase, prevent the biosynthesis of PAR, and induce apoptosis in A549 cells. The same group also reported thaterythrian-triazole F3 (IC_50_ = 0.23–1.13 *μ*M) could exert more potent inhibitory activity than rucaparib (IC_50_ = 2.58–13.82 *μ*M) in a panel of cancer cell lines (A549, OVCAR-3, HepG2, A375, and SW-620) (Li et al., 2020b). Mechanistic studies indicated that F3 could also prevent the biosynthesis of PAR, and induce apoptosis in A549 cells.

Quinine is one of the most abundant natural *cinchona* alkaloids, and also is the mainstay of antimalarial drugs. With the introduction of azide at C9, C2’ and C6’ of quinine, Boratyński et al. synthesized a focused library of triazole containing chinchona alkaloids (F4a, F4b, F5, and F6) (Boratyński et al., 2018). *In vitro* data indicated that nearly all the derivatives could exert moderated antiproliferative activities. Among them, F4a (IC_50_ = 0.53 *μ*M) showed the highest potential in MC-4-11 cells, while, F4b (IC_50_ = 1.2 *μ*M) showed the highest potential in HT-29 cells.

The conjugation of small molecules with ferrocene, a unit that showed tunable redox characteristics, can usually generate new molecules with unexpected properties. Pešić et al. reported that ferrocene-quinine conjugate F8 (IC_50_ = 2.34–2.13 *μ*m) could not only inhibit the growth of drug-sensitive NCI-H460 cancer cells, but also multi-drug resistant (MDR) NCI-H460/R cancer cells (Podolski-Renić et al., 2017). Mechanistic studies indicated that F8 could increase ROS production and induce mitochondrial damage in MDR cancer cells, highlighting the importance of the ferrocene moiety. Panda et al. reported that F7 (IC_50_ = 27 nM) could exert more potent *in vitro* antimalarial activity than quinine (IC_50_ = 58 nM) against *P. falciparum* strain 3D7 (Faidallah et al., 2016), and the reason is probably due to the introduction of the hydrophobic alkyl chain at C9, thereby increasing the penetration ability of the parental scaffold. Sahu et al. reported that C19 quinine-triazole derivative F9 could exert potent antimalarial (*P. falciparum,* IC_50_ = 0.25 *μ*M) and antileishmanial activities (*L. donavani,* IC_50_ = 1.78 *μ*M) with no apparent adverse effects (Sahu et al., 2019). The structural toxicological activity relationship studies indicated that the introduction of the triazole moiety to quinine would result in decreased toxicity.

20(*S*)-Camptothecin is a potent DNA topoisomerase I inhibitor that isolated from *Camptotheca acuminata* in 1966. With the installation of alkyne at C10 of homocamptothecin, followed by reactions with various azides under CuAAC, Xu et al. synthesized a series of C10 homocamptothecin-triazole derivatives. Among them, derivative F10 (IC_50_ = 30 nM) could exert more potent inhibitory than 20(*S*)-camptothecin (IC_50_ = 170 nM) against A549 cancer cells in a Topo I-dependent manner (Xu et al., 2016). Mechanistic studies indicated that F10 could arrest cell cycle at the G2 and S phases.

Colchicine is a well-known antimitotic agent that is isolated from *Colchicum autumnale*. By utilization of the fast and efficient CuAAC derivatization strategy, Schmalz et al. synthesized C7 colchicine-triazole F11, which (IC_50_ = 3.5–5.52 nM) could exert more potent inhibitory activity than colchicine (IC_50_ = 13.2–20.4 nM) in a panel of cancer cell lines (THP-1, Jurkat, Hela, A549 and MES1) (Thomopoulou et al., 2016). In addition, one of the derivative F12 could not only distort the microtubule morphology but also exert a significant centrosome-declustering effect on MDA-MB-231 cells and H1975 cells.

Berberine is an isoquinoline alkaloid that is isolated from various *Berberis* plants. Berberine-triazoles could not only exert anticancer activity but also exhibit antimalarial activity. Sun et al. reported that F13 could inhibit the growth of SW-1990 (IC_50_ = 22.2 *μ*M) and SMMC-7721 (IC_50_ = 14.9 *μ*M) cancer cells (Jin et al., 2014). Nath et al. reported that berberine-triazole F14 (IC_50_ = 0.142 *μ*M) could exert antimalarial activity against *P. falciparum* (3D7) strain with no apparent cytotoxicity in human PC-3 cells (IC_50_ > 200 *μ*g/ml) (Batra et al., 2018).

Hybridization of two different natural products by CuAAC is an efficient strategy to generate novel functional compounds. For example, cytisine-triazole-camphor F15 could exert antiviral activity against influenza virus A/Puerto Rico/8/34 (H1N1) with low toxicity and good selectivity index (IC_50_ = 8 *μ*M, CC_50_ = 168 *μ*M, SI = 20) (Artyushin et al., 2019). Notably, its selectivity index is higher than that of the reference drug rimantadine (IC_50_ = 67 *μ*M, CC_50_ = 335 *μ*M, SI = 5).

Theophylline, a naturally occurring purine base, is a bronchodilator drug that is used for the treatment of various respiratory diseases such as chronic pulmonary obstructive disease and asthma. Murugulla et al. reported that theophylline-triazole F16 could exert potent cytotoxicity on a panel of cancer cells with IC_50_ values in the range of 1.2–2.3 *μ*M (Ruddarraju et al., 2017). In silico docking results indicated that F16 might bind to human epidermal growth factor receptor 2 (EGFR II). Triazole-tethered theophylline-nucleoside hybrid F17 could inhibit the growth of A549, HT-29, MCF-7 and A375 cancer cells (IC_50_ = 1.89–4.89 *μ*m) (Ruddarraju et al., 2016), while hybrid F18 could exert potent antibacterial activities against both Gram-positive (*Staphylococcus aureus*, *Bacillus cereus*) and Gram-negative (*Escherichia coli* and *Pseudomonas aeruginosa*) bacterial strains with MIC values (MIC = 0.03125–0.125 *μ*g/ml), which are comparable to or more potent than that of the clinical drug ciprofloxacin (MIC = 0.0156–0.0625 *μ*g/ml).

## Click Chemistry-Based Modification of Phenylpropanoids

Phenylpropanoids, also known as cinnamic acids, are a series of secondary metabolites that are synthesized by plants from phenylalanine and tyrosine. It mainly includes flavonoids, chalcones, isoflavonoids, lignols, coumarins, stilbenes, aurones, catechin, and phenolic acids.

Flavones are a series of privileged polyphenolic natural products that possess broad-spectrum pharmacological activities. Their synthetic derivatives have been reported to have antitumor, antioxidant, anti-inflammatory, and antiviral activities, etc. Some flavones such as luteolin are under clinical trials for the treatment of cancer (Maggioni et al., 2015), implying the potential of flavones or their derivatives in innovative drug discovery.

**FIGURE 8:**
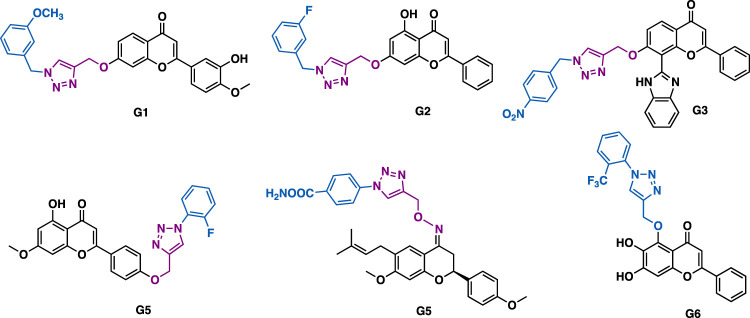
Representative flavone derivatives.

Through the introduction of alkyne functionality at various positions of flavonoids, and the concurrent click chemistry, several series of flavonoid derivatives have been synthesized, though they usually showed moderate biological activities. Patel et al. reported that hesperetin-triazole hybrids G1 (IC_50_ = 14.9–56.8 *μ*M) could exert potent antioxidant activity in DPPH (IC_50_ = 30.6 *μ*M) and ABTS^+^ (IC_50_ = 9.1 *μ*M) assays (Figure 8) (Mistry et al., 2017), and moderate inhibitory activity against a panel of cancer cell lines (CaSki, HeLa and SKOV-3) with low toxicity (Madin-Darby canine kidney (MDCK), IC_50_ = 290.9 *μ*M). Yang et al. reported that chrysin-triazole hybrids G2 (IC_50_ = 1.02–7.30 *μ*M) could exert more potent inhibitory activity than the parental chrysin (IC_50_ = 16.30–73.02 *μ*M) in several cancer cell lines (BEL-7402, HepG-2, SGC-9701) (Luan et al., 2020). Rao et al. reported that the flavone/imidazole-triazole derivative G3 could exert moderate antiproliferative activity against MCF-7 cancer cells (IC_50_ = 17.9 *μ*M) (Rao et al., 2018). Chen et al. reported that apigenin-triazole G4 could exert inhibitory activity against SKOV-3 cells (IC_50_ = 10 *μ*M) (Qi et al., 2018). Mechanistic studies indicated that G4 could promote the level of cellular reactive oxygen species (ROS) and reduce the mitochondrial membrane potential, thereby inducing the apoptosis of SKOV-3 cells. It could also modulate the expression of B-cell lymphoma 2 (Bcl-2) and Bcl-2 associated X protein (Bax). Sangwan et al. reported that bavachinin-triazole G5 (IC_50_ = 30.5–36.3 *μ*M) could exert comparable antiproliferative activity to bavachinin against a panel of cancer cell lines including A549, HCT-116, PC-3, and MCF-7 (Gupta et al., 2018). Mechanistic studies indicated that G5 could induce apoptotic cell death via PARP cleavage and loss of MMP, and it could also inhibit cell migration and colony formation in A549 cells. Baicalein is isolated from *Scutellaria baicalensis*. Niue et al. reported that baicalein-triazole G6 could prevent respiratory tract infection by respiratory syncytial viruses (RSV) via the suppression of oxidative damage (Zhang C. et al., 2019).

Chalcones, also known as chalconoids, are a series of natural polyphenols*.* Structurally, they are *α*, *β*-unsaturated ketones, consisting of two aromatic rings conjugated by an *α*, *β*-unsaturated carbonyl system. Chalcones and their derivatives possess various biological functions such as anticancer, anti-inflammatory and antiviral activities, etc. Thus, hybridization of chalcone scaffold with another pharmacophore by click chemistry may generate derivatives with valuable therapeutic functions.

**FIGURE 9:**
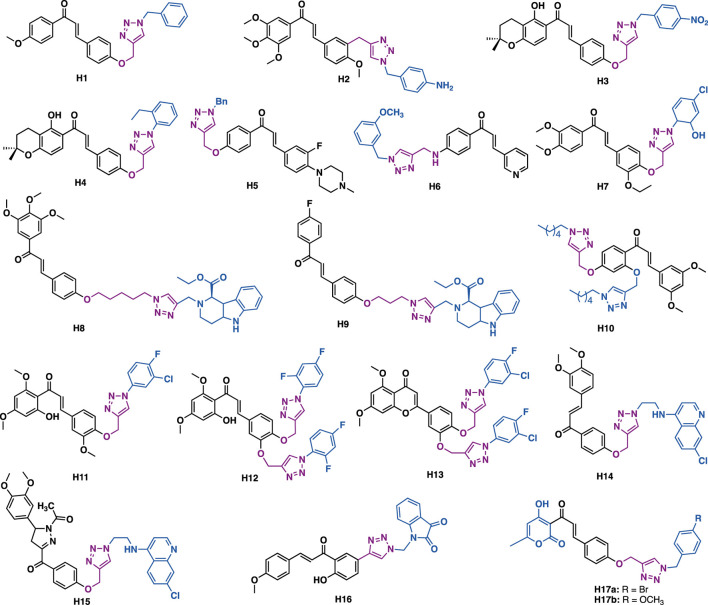
Representative chalcone derivatives.

Lal et al. reported that chalcone-triazole hybrid H1 could exert inhibitory activity against MIA-Pa-Ca-2, MCF-7, HepG2, and A549 cancer cell lines with IC_50_ values in the range of 4–11 *μ*M (Figure 9) (Yadav et al., 2017). Mechanistic studies indicated that H1 could arrest cell cycle (G2/S phase) and induce apoptosis of MIA-Pa-Ca-2 cells by reducing mitochondrial potential and activating PARP-1 and caspase-3.

Kamal et al. reported that triazole-chalcone hybrids H2 could exert low micromolar inhibitory activity against Hela, DU145, HepG2, and A549 cancer cells with IC_50_ values in the range of 1.5–7.7 *μ*M (Hussaini et al., 2016). SAR studies indicated that the *α*, *β*-unsaturated ketone of the chalcone skeleton was critical to the potent cytotoxicity, as the replacement of the *α*, *β*-unsaturated ketone moiety would result in significant loss of cytotoxicity.

Kumar et al. reported that chalcone-triazole hybrid H3 could inhibit the growth of MCF-7, DU-145, and 1MR-32 cancer cells with IC_50_ values in the range of 17.1–29.9 *μ*M (Chinthala et al., 2015), in silico docking studies indicated that H3 might bind to DNA topoisomerase II*α*. While hybrid H4 could exert *α*-glucosidase inhibitory activity (IC_50_ = 67.77 *μ*M), *in silico* docking studies indicated that H4 has a similar binding pattern to the known antidiabetic drug acarbose with *α*-glucosidase (Chinthala et al., 2015). Notably, the 1,2,3-triazole ring might serve as a hydrogen bond acceptor to form two hydrogen bonds with Arg526, and it might also have π−π stacking interaction with Trp406.

Liu et al. reported that hybrid H5 (IC_50_ = 5.47–11.56 *μ*M) and H6 (IC_50_ = 1.53–2.73 *μ*M) could exert low micromolar antiproliferative activity against SK-N-SH, HepG-2, and MGC-803 cancer cells (Fu et al., 2016). Notably, H6 could exert more potent inhibitory activity than 5-fluorouracil (IC_50_ = 7.22–10.32 *μ*M). Mechanistic studies indicated that H6 could arrest cell cycle (G1 phase) and induce apoptosis of SK-N-SH cancer cells.

Subhashini et al. reported that triazole chalcone hybrids H7 could exert submicromolar inhibitory activity against the growth of MCF-7 (IC_50_ = 0.02 *μ*M) and MDA-MB-231 (IC_50_ = 0.31 *μ*M) with low toxicity in non-tumorigenic MCF-10a epithelial cells (IC_50_ = 139.29 *μ*M) (Gurrapu et al., 2020).

Kumar et al. reported that tetrahydro-*β*-carboline-chalcone derivative H8 (IC_50_ = 21.99 *μ*M) could inhibit the growth of MDA-MB-231 cells (Sharma et al., 2020), being three times more potent than the reference drug tamocifen (IC_50_ = 75 *μ*M), while H9 (IC_50_ = 10.33 *μ*M) could inhibit the growth of MCF-3 cells and was five times more potent than tamoxifen. Molecular docking results indicated that they could fit well into the ER*α* ligand binding domain. The analysis of the H9- ER*α* complex indicated that both direct and water-mediated hydrogen bond interactions of triazole and tetrahydro-*β*-carboline with residues (e.g., Leu346, Thr347, and Asp351) crucial to estrogenic inhibitory activity might play a key role in exerting the potent activity (Sharma et al., 2020).

Jalapathi et al. reported that bis-triazole-chalcone hybrid H10 could exert comparable antibacterial activity to the reference drug gentamicin sulphate against several Gram-positive (*Micrococcus luteus, methicillin-resistant Staphylococcus aureus, Bacillus subtilis,* and *Bacillus cereus*) and Gram-negative (*Pseudomonas aeruginosa, Klebsiella pneumoniae, Escherichia coli,* and *Proteus vulgaris*) bacterial strains at concentrations of 75 and 100 *μ*/ml (Sunitha et al., 2020).

Agarwal et al. reported that derivative H11 (IC_50_ = 2.74 *μ*g/ml) could exert potent antiplasmodial activity against the erythrocytic stages of *P. falciparum* (3D7 strain) with no apparent cytotoxicity (Huh-7 cells, CC_50_ > 100 mg/ml) (Kant et al., 2016), while bi-triazole hybrid H12 (MIC = 6.25–12.5 *μ*g/ml) could exert comparable antibacterial activity to ciprofloxacin (MIC = 6.25 *μ*g/ml for all the tested strains) against a panel of bacterial strains (*S. aureus*, *E. faecalis*, *E. coli*, *P. aeruginosa*, *S. boydii,* and *K. pneumoniae*) with low cytotoxicity (Huh-7 cells, CC_50_ > 100 mg/ml) (Kant et al., 2016), and H13 (MIC = 6.25–12.5 *μ*g/ml) could exert potent antifungal activity with low cytotoxicity (Huh-7 cells, CC_50_ > 100 mg/ml) (Kant et al., 2016).

By merging chloroquine (CQ) pharmacophore and chalcone by a triazole linker, Kumar et al. synthesized a potent hybrid derivative H14, which showed potent *in vitro* antiplasmodial activity against *Plasmodium falciparum* (CQR W2 strain, IC_50_ = 114.1 nM) with low cytotoxicity (Hela cells, IC_50_ = 36.5 *μ*M; SI = 311) (Kumar et al., 2017). Further, with the trapping of the *α*, *β*-unsaturated ketone functionality of the hybrid by hydrazine hydrate, followed by acetylation with acetic acid, they have synthesized *N*-acetylpyrazoline derivative H15, which showed increased activity against *Plasmodium falciparum* (CQR W2 strain, IC_50_ = 53.7 nM) and an excellent selectivity index (Hela cells, IC_50_ = 42.7 *μ*M; SI = 795), indicating the *α*, *β*-unsaturated ketone functionality was not critical for antiplasmodial activity.

Hayallah et al. reported that chalcone-triazole-isatin hybrid H16 could exert potent and selective COX-2 inhibitory activity (COX-1, IC_50_ = 13.3 *μ*M; COX-2, IC_50_ = 0.037 *μ*M; SI = 359.46) as well as good 15-LOX inhibitory activity (IC_50_ = 1.95 *μ*M) (Boshra et al., 2020). SAR studies indicated that the isatin moiety would play a key in exerting the potent COX-2 inhibitory activity. *In vitro* anti-inflammatory activity studies indicated that H16 has comparable efficiency as celecoxib at a 3 h interval test.

Lal et al. synthesized a series of dehydroacetic acid chalcone-triazole hybrids, in which the A ring was replaced by dehydroacetic acid (DHA) (Lal et al., 2018). *In vitro* antibacterial and antifungal data indicated that H17a could exert more potent antibacterial activity in both Gram-positive (*Staphylococcus epidermidis,* MIC *=* 0.006 *μ*M/ml; *Bacillus subtilis*, MIC *=* 0.0030 *μ*M/ml) and Gram-negative (*Escherichia coli,* MIC *=* 0.003 *μ*M/ml; *Pseudomonas aeruginosa*, MIC = 0.006 *μ*M/ml) bacterial strains than the reference drug ciprofloxacin (MIC = 0.0047 *μ*M/ml for all the tested strains), while derivative H17b (*Aspergillus niger*, MIC = 0.0068 *μ*M/ml; *Candida albicans*, MIC = 0.0034 *μ*M/ml) could exert more potent antifungal activity than the reference drug fluconazole (*Aspergillus niger*, MIC = 0.0102 *μ*M/ml; *Candida albicans*, MIC = 0.0051 *μ*M/ml).

Coumarins are privileged natural products that possess a fascinating array of biological activities. Their synthetic derivatives have also been reported to exert various pharmacological activities such as anticancer, anti-inflammatory, antibacterial, and antifungal activities, etc.

**FIGURE 10:**
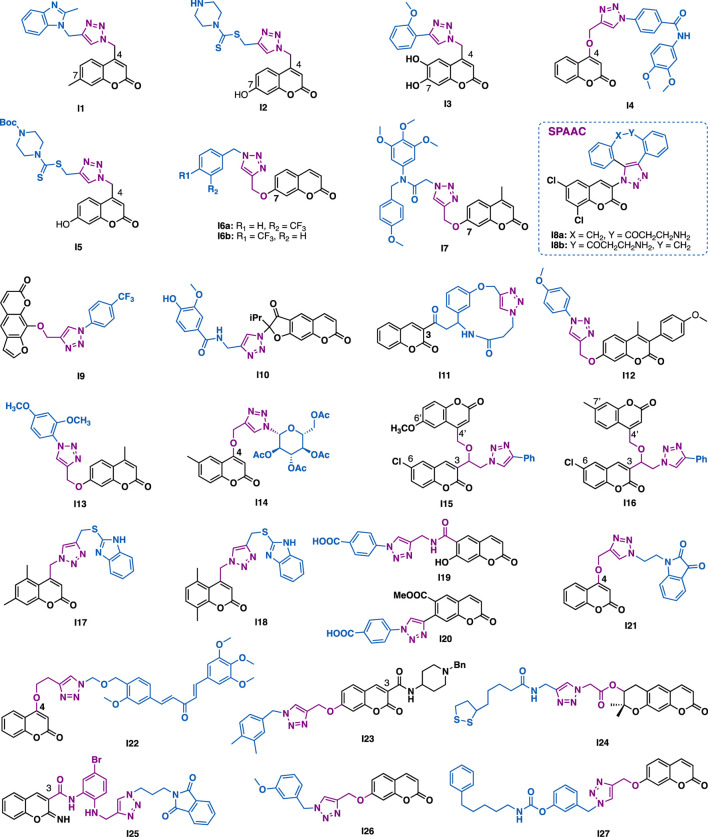
Representative coumarins derivatives.

Raić-Malić et al. reported that C4 coumarin-triazole I1 could exert submicromolar inhibitory activity against HepG2 cells (IC_50_ = 0.9 *μ*M) with low toxicity (normal fibroblasts WI38, IC_50_ = 45.33 *μ*M, SI = 50) (Figure 10) (Kraljević et al., 2016). Mechanistic studies indicated that I1 could inhibit 5-lipoxygenase (5-LO), disturb intracellular acid ceramidase (ASAH) activity, thereby perturbing sphingolipid signaling, while I2 could exert high selectivity antibacterial activity against *Enterococcus species* (MIC = 16 *μ*g/ml). Moreover, it could also inhibit the growth of clinically derived vancomycin-resistant *Enterococcus faecium* (MIC = 64 *μ*g/ml), whereas the reference antibiotics ceftazidime (CAZ) and ciprofloxacin (CIP) were inactive (MIC >256 *μ*g/ml) (Kraljević et al., 2016). The same group further reported C4 modified derivative I3 could exert potent antioxidant activity (DPPH assay), and moderate cytotoxic activity against Hela, CaCo-2, and K562 cancer cells (IC_50_ = 9.7–41.6 *μ*M) (Bistrović et al., 2017).

Guo et al. reported that the ether tethered triazole-coumarin I4 could potently inhibit the growth of MDA-MB-231 cells under hypoxia conditions (IC_50,*hypoxia*_ = 0.03 *μ*M; IC_50,*normoxia*_ = 1.34; SI_*hypoxia/normoxia*_ = 46.31) (An et al., 2018), and it was 20 times and 156 times more potent than that of doxorubicin (IC_50_ = 0.6 *μ*M) and cisplatin (IC_50_ = 4.68 *μ*M), respectively, whereas the parental 4-hydroxycoumarin was inactive at the concentration of 100 *μ*M.

Liu et al. reported that C4 coumarin derivative I5 could inhibit the growth of PC-3, MGC-803, and MCF-7 cancer cells (IC_50_ = 4.96–36.84 *μ*M), and that was comparable to or more potent than that of 5-fluorouracil (IC_50_ = 7.01–27.07 *μ*M) (Duan et al., 2013).

Magolan et al. reported that C7 coumarin derivative I6a could inhibit the growth of pancreatic cancer cell lines including MIA PaCa-1, Capan-1, and PANC-1 (IC_50_ = 8.5–29 *μ*M). A subtle change of the position of the trifluoromethyl from *meta*-to *para*-position (I6b) led to the inactivity of the compound against Capan-1 and PANC-1 cells (IC_50_ > 100 *μ*M), but slightly increased cytotoxic activity against MIA PaCa-1 cells (IC_50_ = 9.6 *μ*M), implying the trifluoromethyl may play a key role in exerting the selective inhibitory activity against MIA PaCa-1 cells (Farley et al., 2016).

Zhang et al. reported that C7 modified hybrid I7 (PC-3, IC_50_ = 0.34 *μ*M; MGC803, IC_50_ = 0.13 *μ*M) could exert more potent inhibitory activity than colchicine (PC-3, IC_50_ = 0.59 *μ*M; MGC803, IC_50_ = 0.27 *μ*M) against PC3 and MGC803 cancer cells (Fu et al., 2019). Mechanistic studies indicated that I7 could arrest cell cycle (G2/M phase), inhibit colony formation, and promote apoptosis of the tested MGC803 cells by regulating Bcl-2 and DR5. In addition, I7 (1 *μ*M) could inhibit tubulin polymerization by interacting with the colchicine binding site.

By using the SPAAC click chemistry of cyclooctyne (DBCO) and azide-coumarin, Paira et al. synthesized I8a and its regio-isomer I8b with the aim of theranostic application (Sinha et al., 2016). *In vitro* data indicated that both I8a and I8b could exhibit maximum quantum yields and good uptake by MCF-7 cells, implying their potential for cancer diagnosis. Moreover, they could inhibit the growth of Hela (IC_50_ = 17.5 *μ*M) and MCF-7 (IC_50_ = 9.83 *μ*M) cancer cells with good selectivity.

Xanthotoxin is a furanocoumarin identified from the traditional Egyptian medicinal plant *Ammi majus L*. Quan et al. reported that xanthotoxin-triazole I9 could exert potent antiproliferative activity against AGS cells (IC_50_ = 7.5 *μ*M) with low toxicity (normal L02 cell, IC_50_ > 100 *μ*M) (Shen et al., 2017), and that it was more potent than the parental xanthotoxin (IC_50_ > 100 *μ*M) and the reference drug 5-fluorouracil (IC_50_ = 29.6 *μ*M).

Shults et al. reported that reoselone-triazole I10 could inhibit the growth of CEM-13, U-937, and MT-4 cancer cells with IC_50_ values in the range of 8–10 *μ*M (Lipeeva et al., 2015). Molecular docking studies indicated that I10 might bind to the active site of phosphodiesterase (PDE-4B) and showed good interactions with amino acid residues of PDE4B. Both the aryl-substituted triazole and the dihydrofurocoumarin were involved in the π−π stacking interaction with Phe446 (Lipeeva et al., 2015). Notably, the triazole ring of I10 might involve in the binding with sulfurs of Met347 and Met431 by forming π−sulfur interactions. Bahulayan et al. reported that some coumarin-containing macrocyclic derivatives, like I11, could also exert inhibitory activity against cancer cells (Raj and Bahulayan, 2017).

Dharavath et al. reported that I12 (IC_50_ = 1.29 *μ*M) could exert comparable antioxidant activity to ascorbic acid (IC_50_ = 1.46 *μ*M) in DPPH assays (Dharavath et al., 2020). *In vitro* anti-inflammatory data indicated that I12 (IC_50_ = 15.90 *μ*M/ml) could exert more potent activity than the reference drug diclofenac (IC_50_ = 17.52 *μ*M) in heat-induced hemolytic assays. The antibacterial activity evaluation results indicated that I12 could inhibit the growth of the tested Gram-positive (*Staphylococcus aureus* and *Bacillus subtilis*) and Gram-negative (*Escherichia coli* and *Klebsiella pneumonia*) bacterial strains at the concentrations of 10 or 20 *μ*g/ml. Moreover, I12 could also exert antifungal activity against three fungal strains (*Aspergillus niger*, *Aspergillus favus,* and *Fusariumoxy sporum*) at the concentration of 50 *μ*g/ml, and it was comparable to the reference drug clotrimazole.

Awasthi et al. reported that coumarin-triazole I13 could exert potent antimalarial activity against *P. falciparum* 3D7 strain (IC_50_ = 0.763 *μ*g/ml) with low cytotoxicity (human hepatoma cell (huh7), CC_50_ > 100 *μ*g/ml) (Yadav et al., 2018). Mechanistic studies using *Escherichia coli* DNA gyrase, indicated that I13 might disrupt the catalytic activity of DNA enzyme gyrase, thereby switching off its supercoiling activity.

Sagar et al. reported that triazole-*N*-glycoside-coumarin I14 could exert low micromolar (IC_50_ = 10.97 *μ*M) selective inhibitory activity against breast MCF-7 cancer cells (Kumari et al., 2019). Mechanistic studies indicated that I14 could promote the level of cellular reactive oxygen species (ROS), thereby inducing the generation of toxic products in MCF-7 cancer cells.

Kalkhambkar et al. reported that derivative I15 (MIC = 1 *μ*g/ml) could exert comparable antibacterial activity to the reference drug ciprofloxacin (MIC = 1 *μ*g/ml) in the tested Gram-positive (*Staphylococcus aureus*) and the Gram-negative (*Pseudomonas aeruginosa*) bacterial strains (Savanur et al., 2018), while I16 could exert comparable antifungal activity to itraconazole against *Candida albicans* and *Aspirgillus niger* strains with MIC values of 1 *μ*g/ml.

Kulkarni et al. reported that coumarin-2-mercaptobenzimidazole hybrid I17 (MIC = 3.8 *μ*M) and I18 (MIC = 3.8 *μ*M) could exert more potent antibacterial activity than the reference drugs pyrazinamide (MIC = 25.2 *μ*M), streptomycin (MIC = 10.7 *μ*M), and ciprofloxacin (MIC = 9.4 *μ*M) against *M. tuberculosis* (H_37_Rv) (Anand et al., 2016).

Shults et al. reported that I19 (*S. aureus* 209p, MIC = 0.16 *μ*M; *S. aureus* C-18, MIC = 0.65 *μ*M) could exert 6- and 10-fold more potent antibacterial activity than the reference drug ceftriaxone (*S. aureus* 209p, MIC = 0.97 *μ*M; *S. aureus* C-18, MIC = 6.5 *μ*M) against *Staphylococcus aureus* 209p and C-18 bacterial strains (Lipeeva et al., 2019), while I20 (MIC = 0.21 *μ*M) could exert a 5-fold more potent antibacterial activity than ceftriaxone (MIC = 1.03 *μ*M) against *Staphylococcus aureus* “Viotko” bacterial strain.

Bedi et al. reported that coumarin-isatin I21 could exert promising antiproliferative activity against THP-1, COLO-205, and HCT-116 cancer cells (IC_50_ = 0.73–3.45 *μ*M) by inhibiting the polymerization of the tubulin (IC_5**0**_ **=** 1.06 *μ*M) (Singh et al., 2017). The same group also reported that coumarin-curcuminoid hybrid I22 could exert promising antiproliferative activity against THP-1, COLO-205, and HCT-116 cancer cells (IC_50_ = 0.82–4.68 *μ*M) by inhibiting the polymerization of the tubulin (IC_50_ **=** 1.55 *μ*M) (Singh et al., 2016).

For a long time, the great potential of coumarin-triazole hybrids in anti-Alzheimer’s disease drug discovery has been well demonstrated by the target-based screening of acetylcholinesterase (AChE) (Saeedi et al., 2017; Rastegari et al., 2019), butyrylcholinesterase (BuChE) (Park et al., 2016), and *β*-secretase (BACE1) inhibitors (Iraji et al., 2017). For example, Saeedi et al. reported that I23 could exert selective AChE inhibitory activity (AChE, IC_50_ = 1.8 *μ*M; BuChE, IC_50_ > 100 *μ*M) (Rastegari et al., 2019). And also, it could exert a neuroprotective effect against H_2_O_2_-induced cell death of PC12 neurons. Park et al. reported that decursinol-lipoic acid-triazole hybrid I24 (AChE, IC_50_ > 350 *μ*M; BuChE, IC_50_ = 5.89 *μ*M) could exert selective BuChE inhibitory activity (Park et al., 2016), and that it is more potent than that of galantamine (BuChE, IC_50_ = 9.4 *μ*M). SAR studies indicated that its selectivity (AchE/BuChE) might result from neither decursinol nor triazole, but the hybrid derivative I24. *β*-Secretase (BACE1), a transmembrane aspartic protease, is an attractive target for the development of AD therapeutic drugs as it is critical in trigging the amyloidogenic pathway. Miri et al. reported that iminochromene-triazole I25 (Iraji et al., 2017), a non-peptidic *β*-secretase (BACE1) inhibitor, could exert promising BACE1 inhibitory activity (IC_50_ = 2.2 *μ*M) with no apparent cytotoxicity. Notably, it could exert a 10-fold more potent neuro protective effect (IC_50_ = 7.9 *μ*M) than the reference caffeic acid (IC_50_ = 75.8 *μ*M) in A*β* induced toxicity in pC12 neurons. Foroumadi et al. synthesized a dual AChE (IC_50_ = 3.4 *μ*M) and BuChE (IC_50_ = 1.1 *μ*M) inhibitor I26 (Moradi et al., 2018), which could also exert promising protective effective in H_2_O_2_-induced cell death of PC_12_ neurons. With the aim of discovering a multi-target-directed ligand for neurodegenerative disease, Rampa et al. synthesized I27 (Montanari et al., 2016), a dual FAAH/BuChE inhibitor, which could exert well-balanced nanomolar inhibitory activities (*r*FAAH, IC_50_ = 27.9 nM; *h*BuChE, IC_50_ = 42.7 nM; hAChE, IC_50_ = 922 nM). Considering that indirectly enhancing endocannabinoid signaling by FAAH inhibitors might be preventing or slowing the progression of neurodegenerative disorders such as Alzheimer’s disease, I27 may be a valuable candidate for AD treatment.

**FIGURE 11:**
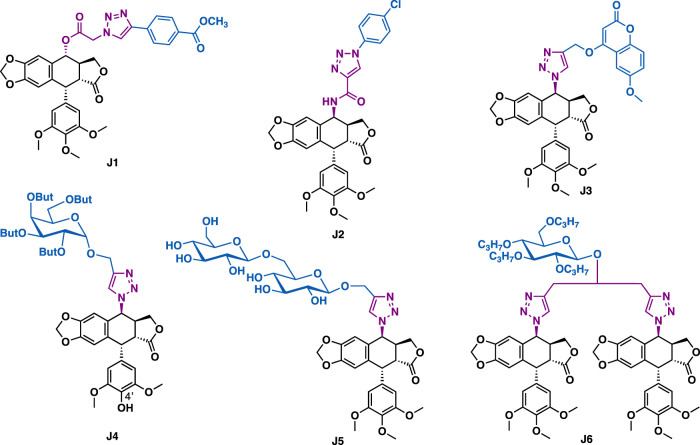
Representative podophyllotoxin derivatives.

Podophyllotoxin is a natural lignin that is isolated from the roots of *Podophyllum hexandrum*. Podophyllotoxin and its derivatives could exert antiproliferative activity by inhibiting tubulin polymerization, while epipodophyllotoxins and its derivatives could inhibit topoisomerase II. So far, some podophyllotoxin-/epipodophyllotoxin-derived therapeutic agents such as teniposide and etoposide have already entered into clinical use for the treatment of cancer. Diversification of podophyllotoxin by click chemistry has been reported to generate derivatives with increasing inhibitory activity whereas reducing toxicity. For example, 4*α*-podophyllotoxin-triazole J1 could exert broad-spectrum inhibitory activity against a panel of cancer cell lines (A549, PC-3, MCF-7, U251, SKBR-3, and LNCaP) with IC_50_ values in the range of 19.6–42.9 nM (Figure 11). Moreover, it could effectively overcome drug resistance, and showed weak cytotoxicity in non-cancer cells. Preliminary mechanistic studies implied that J1 could interact with microtubule, arrest cell cycle (G2/M phase) and induce cell apoptosis in PC-3 cells (Hou et al., 2019c). Kamal et al. reported that epipodophyllotoxin-triazole J2 (IC_50_ = 0.70–4.11 *μ*M) could exert potent inhibitory activity against a panel of cancer cell lines (A549, MCF-7, DU-145, Hela, HepG2, and HT-29) with weak cytotoxic activity in normal NIH/3T3 cells (IC_50_ = 89.04 *μ*M) (Reddy et al., 2018). Mechanistic studies indicated that J2 could inhibit topoisomerase II, arrest cell cycle (G2/M phase), and effectively induce apoptosis of the tested DU-145 cells. Hui et al. reported that podophyllotoxin-triazole-coumarin hybrid **J3** (IC_50_ = 4.9–17.5 *μ*M) could exert more potent inhibitory activity than the etoposide (IC_50_ = 10.5–25.6 *μ*M) in a panel of cancer cell lines including A549, HepG2, HeLa, and LoVo (Hao et al., 2019). Mechanistic studies indicated that **J3** could bind to CT DNA, disrupt microtubules, arrest cell cycle (G1 phase) and inhibit Topo-II *β*.

Podophyllotoxin-triazole-sugar hybrids also possess promising inhibitory activity against various cancer cells. For example, Jiang et al. reported that hybrid J4, which bearing a perbutylated *α*-D-(+)-galactosyl residue could exert promising inhibitory activity against a panel of cancer cell lines (A-549, HL-60, MCF-7, SMMC-7721, and SW480) with IC_50_ values in the range of 0.49–6.70 *μ*M, (Zi et al., 2015), which is more potent than or comparable to the reference drugs etoposide and cisplatin. SAR studies indicated that the spacer between triazole and sugar residue, as well as a 4'- hydroxyl group of podophyllotoxin scaffold that might play a key role in exerting the potent activity. Derivative J5, which bearing a 1,6-*β*-D-di-glucose residue could exert potent inhibitory activity against the tested HL-60, SMMC-7721, A549, MCF-7, and SW480 cancer cells with IC_50_ values in the range of 0.67–7.41 *μ*M (Zi et al., 2017). Hu et al. reported that bis-triazole-tethered bis-epipodophyllotoxin-glucose J6 (IC_50_ = 0.43–3.50 *μ*M) could exert more potent inhibitory activity than cisplatin (IC_50_ = 1.67–10.85 *μ*M) in several cancer cell lines including HL-60, SMMC-7721, A549, MCF-7 and SW480 with low toxicity (normal BEAS-2B cells, IC_50_ = 15.38 *μ*M; SI = 4.4–35.8) (Zi et al., 2018).

**FIGURE 12:**
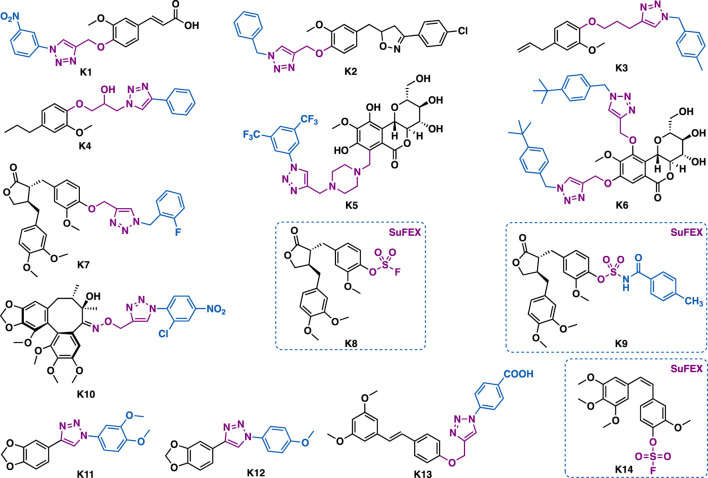
Representative phenylpropanoids K1-K14.

Ferulic acid is an abundant phenolic phytochemical found in plant cell walls. Abid et al. reported that ferulic acid-triazole K1 could exert selective inhibitory activity against carbonic anhydrase IX (CA IX) (IC_50_ = 24 nM) (Figure 12) (Aneja et al., 2020). Further studies indicated that K1 could inhibit colony formation and cell migration, downregulate CA IX expression, decrease epithelial to mesenchymal transition (EMT), and induce apoptosis in HepG2 cancer cells.

Eugenol is the principal active component of clove oil. Eugenol-triazoles possess several biological activities such as anticancer and anti-parasitic activities. Morjani et al. reported that eugenol-triazole K2 could exert broad-spectrum antiproliferative activity against a panel of cancer cell lines including HT1080, A549, MCF-7, and MDA-MB-231 with IC_50_ values in the range of 15.31–23.51 *μ*M (Taia et al., 2020). Teixeira et al. reported that eugenol-triazole K3 could not only exert extracellular leishmanicidal activities (IC_50_ = 7.4 *μ*M) (Teixeira et al., 2018), but also intracellular leishmanicidal activities against leishmania parasites inside peritoneal macrophages (IC_50_ = 1.6 *μ*M) without interfering with the viability of macrophages (IC_50_ = 211.9 *μ*M; SI = 132.5). Notably, it was more potent than clinical drugs glucantime and pentamidine. De Souza et al. reported that dihydroeugenol-triazole K4 (IC_50_ = 42.8 *μ*M) could exert comparable trypanocidal activity to the reference drug benznidazole against the epimastigote forms of *Trypanosoma cruzi* (*T. cruzi.,* Y strain) with low toxicity (Souza et al., 2020). *In vivo* data showed that K4 (100 mg/kg, p. o.) could reduce more than 50% of the parasitemia in *T. cruzi* infected mice*.*

Bergenin, a dihydroisocoumarin, has been reported to have various biological activities such as anti-HIV, neuroprotective, and anticancer activities (Bajracharya, 2015). Babu et al. reported that derivative K5 could inhibit the growth of A549 (IC_50_ = 1.86 *μ*M) and HeLa (IC_50_ = 1.33 *μ*M) cells (Pavan Kumar et al., 2019), and that was comparable to doxorubicin. Mechanistic studies indicated K5 could arrest cell cycle (G2/M phase) and induce apoptosis in Hela cells. Moreover, it could inhibit the polymerization of tubulin and disrupt the balance of intracellular tubulin-microtubule. Yang et al. reported that K6 (IC_50_ = 6.2–17.6 *μ*M) could exert more potent inhibitory activity than the parental bergenin against EC9706, B16 and MGC803 cancer cells (Yang et al., 2015). In addition, it (IC_50_ = 6.2 *μ*M) could exert comparable inhibitory activity to 5-fluorouridine (IC_50_ = 6.3 *μ*M) in EC9706 cancer cells.

Arctigenin is a lignan that is isolated from the dry ripe fruit of *Arctium lappa*. Quan et al. reported that arctigenin-triazole K7 could exert more potent and selective anti-*Toxoplasma gondii* activity (*Toxoplasma gondii*, IC_50_ = 17.1 *μ*M; Hela, IC_50_ = 600 *μ*M; SI = 35.09) than both of the lead arctigenin (*Toxoplasma gondii*, IC_50_ = 586.4 *μ*M; Hela, IC_50_ = 572.7 *μ*M; SI = 0.98) than the reference drug spiramycin (*Toxoplasma gondii*, IC_50_ = 262.2 *μ*M; Hela, IC_50_ = 189.0 *μ*M; SI = 0.72).(Zhang H.-b. et al., 2018). By using sulfur (VI) exchange chemistry, Zhang et al. synthesized derivatives K8 and K9 (Zhang S. et al., 2019), and preliminary *in vitro* data indicated that these compounds could exert good anti-inflammatory activity.

Babu et al. reported that dibenzoclooctene type natural product gomisin B-triazole K10 could exert broad-spectrum antiproliferative activity against a panel of cancer cell lines including A549, DU-145, MDA-MB-231, PANC1, IMR32, and SIHA cells (IC_50_ = 0.24–12.8 *μ*M). Particularly, it could exert submicromolar inhibitory activity against SIAH cells (IC_50_ = 0.24 *μ*M), (Poornima et al., 2017), and it was more potent than that of the parental Gomisin B (IC_50_ = 51.2–66.8 *μ*M). Mechanistic studies indicated that it could stall cell cycle (G2/M phase) and promote tubulin polymerization in the tested HeLa cells.

Machilin G is a natural lignan that is isolated from *Magnolia denudate*. Replacement of the tetrahydrofuran ring of machilin with 1,2,3-triazole, the resulting mimic K11 (IC_50_ = 1.1 *μ*M) could exert 8-fold more potent inhibitory activity than the recommended drug pentamidine (IC_50_ = 8.9 *μ*M) against promastigote form of *L. amazonensis* (Cassamale et al., 2016)*.* Moreover, it (NIH/3T3, IC_50_ = 768.5 *μ*M; SI = 698.6) showed a higher selectivity index than that of pentamidine (NIH/3T3, IC_50_ = 78.7 *μ*M; SI = 8.8), while derivative K12 could exert selective inhibitory activity against *Trypanosoma cruzi* trypomastigotes with an IC_50_ value of 28.6 *μ*M and SI value of 29.6.

Pterostilbene is a bioactive natural stilbenoid that is isolated from blueberries and *Pterocarpus marsupium* heartwood. Structurally, it is very similar to resveratrol, a healthy benefiting compound that is rich in red wine. Diversification of pterostilbene by CuAAC could generate derivatives with improved antibacterial activity. For example, derivative K13 could exert potent antibacterial activity against methicillin-resistant *Staphylococcus aureus* (MRSA) with an MIC value of 1.2–2.4 *μ*g/ml and a minimum bactericidal concentration (MBC) of 19.5–39 *μ*g/ml, (Tang et al., 2019), while the MIC value of pterostilbene is 41–161.5 *μ*g/ml. Mechanistic studies indicated that it could inhibit DNA polymerase, but not the bacterial cell membrane and cell wall.

Combretastatin A-4 (CA-4) is a stilbenoid phenolic natural product that is isolated from the African willow tree, *Combretum caffrum* (Shan et al., 2011). It can exert potent reversible inhibitory activity in the polymerization of tubulin. Structural modifications of CA-4 have yielded several novel CA-4 derivatives with potent tubulin inhibitory activity. Taking advantage of the powerful sulfur (IV) fluoride exchange (SuFEX) click chemistry, Wu et al. synthesized the fluorine sulfonate CA-4 K14 (IC_50_ = 8.9 *μ*M), which could exert 70-fold more potent inhibitory activity than the parental CA-4 against drug in HT-29 cells (IC_50_ = 8.9 *μ*M) (Liu et al., 2018).

## Click Chemistry-Based Modification of Steroids

Steroids are series of naturally occurring compounds that are ubiquitously distributed in animals, plants and fungi, etc. They can act as signaling molecules or as key components of cell membranes. Derivatization of steroids by click chemistry can quickly generate novel molecules with new functions for drug discovery.

**FIGURE 13:**
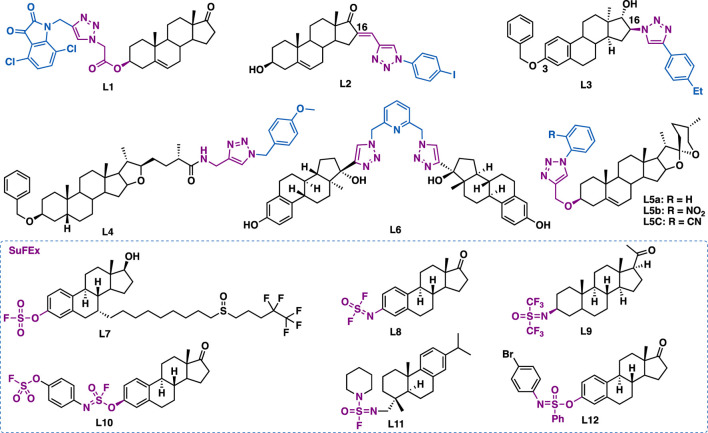
Representative steroids derivatives.

Dehydroepiandrosterone is a unique active substance found in sweet potato and yam. Dehydroepiandrosterone derivatives containing triazole at the C3 and/or C16 could exert antiproliferative effects. By molecular hybridization of dehydroepiandrosterone and isatin, Liu et al. synthesized hybrid L1 (Figure 13) (Yu et al., 2016), which could exert comparable inhibitory activity (IC_50_ = 4.06 *μ*M) to the reference drug 5-fluorouracil (IC_50_ = 3.26 *μ*M) in SH-SY5Y cancer cells. Mechanistic studies indicated that L1 could potently inhibit LSD1 (C_50_ = 3.18 *μ*M), decrease mitochondrial membrane potential, arrest cell cycle (G2/M phase), and induce cell apoptosis. Notably, L1 is the first steroid-based lysine-specific demethylase (LSD1) inactivator. Quan et al. reported that C16 dehydroepiandrosterone-triazole L2 could inhibit the growth of HepG-2 (IC_50_ = 9.18 *μ*M) and MCF-7 (IC_50_ = 9.18 *μ*M) cancer cells by arresting cell cycle (G2 phase) and inducing cell apoptosis (Huang X. et al., 2018). Mernyák et al. reported that C16 *α*-estrone-triazole L3 (IC_50_ = 2.6–6.5 *μ*M) could exert broad-spectrum antiproliferative activities against a panel of cancer cell lines including HeLa, MCF-7, A431, A2780, T47D (expressing androgen, progesterone and estrogen receptors), MDA-MB-231 (expressing HER2 and estrogen receptor) and triple-negative MDA-MB-361 (Mernyák et al., 2015), and it was comparable to or better than the reference drug cisplatin (IC_50_ = 1.3–19.1 *μ*M). Mechanistic studies indicated that L3 could induce apoptosis by the intrinsic pathway.

Sarsasapogenin is one of the active ingredients that is isolated from *Rhizoma anemarrhenae*. Song et al. reported that sarsasapogenin-triazole L4 could inhibit the aggregation of A*β*_1-42_ (IC_50_ = 5.84 *μ*M) (Wang W. et al., 2018). Moreover, *in vitro* data indicated that L4 could exert moderate neuroprotective effects against H_2_O_2_-induced neurotoxicity in SH-SY5Y cells. Further *in vivo* studies showed that L4 (17.5 mg/kg, p. o.) could significantly ameliorate cognitive impairments in behavioral tests, and it was comparable to or better than the reference drug cisplatin.

Diosgenin, is a steroidal sapogenin that isolated from *Dioscorea deltoidei*. Structurally, it is similar to cholesterol and other steroids. Ara et al. reported that diosgenin-triazole hybrids L5a (IC_50_ = 5.54–10.33 *μ*M), L5b (IC_50_ = 5.77–8.67 *μ*M) and L5c (IC_50_ = 6.33–9.44 *μ*M), bearing simple phenyl moiety at the C4’ of the triazole moiety, could inhibit the growth of A549, HCT-116, HT-29, and HBL-100 cancer cell lines (Masood-ur-Rahman et al., 2017).

Sedlák et al. reported the triazole tethered estradiol dimers L6 (IC_50_ = 0.49–3.65 *μ*M) could exert potent inhibitory activity in a panel of cancer cell lines including A549, HeLa, HCT116, K562, K562-Tax (Paclitaxel resistant), CCRF-CEM (childhood T acute lymphoblastic leukemia), U2OS, and HCT116p53/(null p53 gene) by inhibiting tubulin polymerization (Jurášek et al., 2018).

Taking advantage of the emerging SuFEx click chemistry, several steroid derivatives such as fluorosulfate L7 (Liu et al., 2018), iminosulfur oxydifluoride L8 (Li S. et al., 2017), bis(trifluoromethyl)sulfur oxyimine L9 (Smedley et al., 2019), sulfurofluoridoimidate L10 (Li S. et al., 2017), sulfonimidoyl fluoride L11 and sulfonimidate L12 have been synthesized by using SO_2_F_2_ or its sister gas SOF_4_ as key reagents in the presence of tertiary amine additives (e.g., TEA, DIPEA, DBU) (Li S. et al., 2017; Gao et al., 2018; Smedley et al., 2019). Among them, the fluorosulfate version of fulvestrant L7 (IC_50_ = 4.8–5.5 nM) could exert more potent inhibitory activity than fulvestrant (IC_50_ = 7.7–14.8 nM). Notably, its inhibitory activity was ER^–^ dependent (MCF7, IC_50_ = 5.5 nM; ER^–^ MCF7, IC_50_ > 10,000 nM). The biological activities of the other SuFEx click chemistry steroid derivatives are yet to report.

## Click Chemistry-Based Modification of Xanthones and Quinones

**FIGURE 14:**
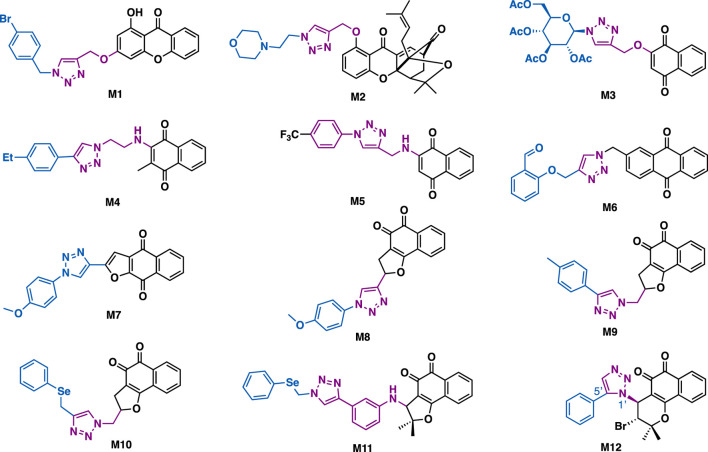
Representative xanthone and quinone derivatives.

Xanthones is a series of bioactive substance that can be readily obtained from plants and/or microorganisms. The key structural feature of these compounds is a biphenyl pyranone containing a planar three-ring system. Yu et al. reported that M1 could exert inhibitory activity against A549 cells (IC_50_ = 32.4 *μ*M) (Wu et al., 2019). Western blotting data indicated that M1 could significantly upregulate protein levels of caspase 3, Bax, c-Jun N-terminal kinase, and also p53 in A549 cells (Figure 14). Zhang et al. reported that gambogic acid-triazole M2 (IC_50_ = 0.31–3.79 *μ*M) could exert sustained cytotoxicity against a panel of cancer cell lines (U2OS, HepG2, A549, and HCT116) and two drug resistant cancer cell lines (Taxol-resistant or cisplatin-resistant A549 cells) with improved aqueous solubility and permeability (Li X. et al., 2017). Notably, it could exert *in vivo* antitumor activity in A549-transplanted mice models.

Quinone is a privileged pharmacophore that presents in many bioactive natural products, such as mitomycin, saintopin, and doxorubicin. Its various promising biological activities might attribute to i) its ability to generate ROS, which usually leads to the damage of DNA, and ii) its ability to electrophilic arylation of critical cellular nucleophiles. Thus, the derivatization of quinone by using click chemistry would quickly generate molecules with desirable functions.

Lawsone is a natural bioactive quinone that is isolated from *genus Lawsonia*. Alves et al. reported that Lawsone-glycosyl triazole M3 could exert promising inhibitory against SKBR-3 cells (IC_50_ = 0.78 *μ*M) with good selectivity index (normal HGF cell, IC_50_ = 17.65 *μ*M, SI = 22.6) (Ottoni et al., 2020). It is more potent than lawsone (IC_50_ > 50 *μ*M), which could be ascribed to the introduction of the peracetylated D-glucose to the hybrid M3, thereby generating a more favorable lipophilic–hydrophilic balance and being absorbed by tumor cells more easily.

Naphthoquinone-triazole hybrid M4 could exert inhibitory activity against DU-145, Hela, A549, and MCF-7 cancer cell lines (IC_50_ = 8.02–26.12 *μ*M), (Prasad et al., 2018), and that was comparable to the reference drug tamoxifen (IC_50_ = 10.87–18.63 *μ*M). Mechanistic studies implied that M4 could arrest cell cycle (G0/G1 phase) and induce apoptosis in MCF-7 cancer cells. While, naphthoquinone-triazole M5 (IC_50_ = 6.8–10.4 *μ*M) could exert broad-spectrum inhibitory activity against HT-29, MOLT-4, and MCF-7 cancer cell lines (Gholampour et al., 2019), and could arrest cell cycle (G0/G1 phase) in the tested MCF-7 cells.

Anthraquinone has the structural core of anthracycline. Meng et al. reported that anthraquinone-triazole derivative M6 (IC_50_ = 0.6 *μ*M) could exert more potent inhibitory against xanthine oxidase_ a well-known target for the treatment of hyperuricemia and gout, than the reference allopurinol (IC_50_ = 9.8 *μ*M) (Zhang et al., 2017). SAR studies revealed that the benzaldehyde moiety might play a more important role than the anthraquinone moiety in its inhibitory potency.

The 1,4-furanaphthoquinone-triazole hybrid M7 (IC_50_: 81.81–99.56 *μ*M) and its regioisomer 1,2-furanaphthoquinone-triazole hybrid M8 (IC_50_: 23.04–41.10 *μ*M) could only exhibit moderate inhibitory activity against MDA-MB-231 and CaCo-2 cancer cells (Costa et al., 2018), whereas 1,2-furanaphthoquinone-triazole M9 (IC_50_: 0.74–1.77 *μ*M) could exert superior cytotoxic activity against HCT-116 and MCF-7 cancer cells (Chipoline et al., 2018), and the selenium version hybrid M10 (IC_50_: 0.07–0.29 *μ*M) and M11 (IC_50_: 0.07–0.38 *μ*M) showed high activity against HL-60, PC3, HCT-116, SF295, OVCAR-8, and MDA-MB-435 cancer cell lines (da Cruz et al., 2016). Mechanistic studies revealed that their apoptosis effect was associated with ROS production. The 1,2-naphthoquinone-triazole M12 (IC_50_ = 0.41–1.59 mM) bearing an pyran fragment also showed good inhibitory potency against PC3, HL-60, SF-295, HCT-116 and MDA-MB-435 cancer cells (Bahia et al., 2016).

## Click Chemistry-Based Modification of Macrocyclic Natural Products

**FIGURE 15:**
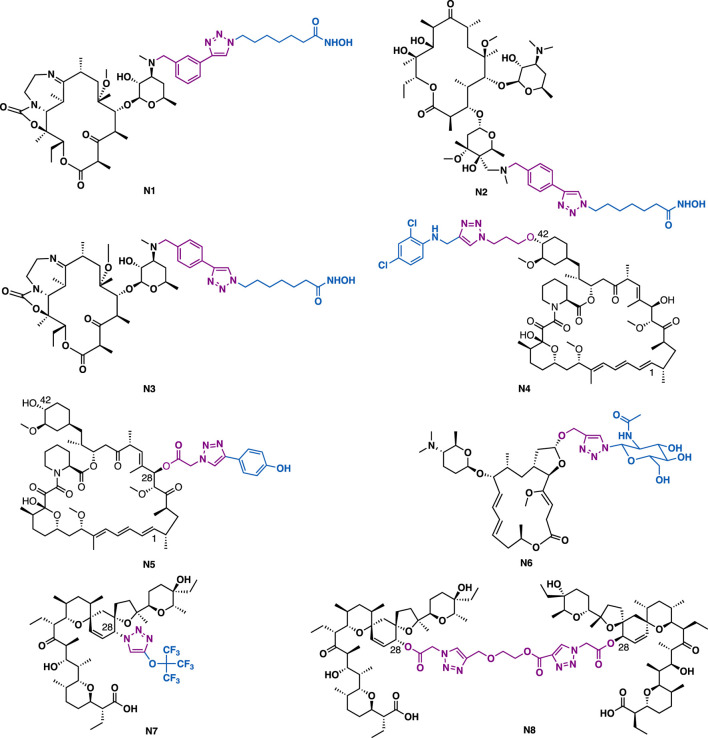
Representative macrocyclic derivatives.

Peptidic macrocyclic histone deacetylases inhibitors (HDACi) contain diverse cap groups that are capable of producing the optimal interactions with amino acid residues that surround the entrance of the HDAC active site, thereby modulating the biological activities of these HDACis. Although they usually could exert nanomolar inhibitory activity against HDACs, their further clinical development has been hindered greatly due to the difficulty in the synthesis of cyclic peptide frameworks for SAR studies. With the aim to solve these drawbacks, Oyelere et al. chose 14-membered macrolide clarithromycin to mimic the peptidic framework of macrocyclic HDACis’. The results indicated that macrolide is a good mimic of the peptide framework. For example, hybrids N1, N2, and N3, could exert moderate HDAC-6 inhibitory activity with IC_50_ values of 3.76, 2.85, and 6.99 *μ*M (Figure 15) (Tapadar et al., 2015), respectively. Hybrid N1 (IC_50_ = 0.86 *μ*M) and N2 (IC_50_ = 0.69 *μ*M), both bearing a zinc chelating hydroxamate moiety, could exert potent antiproliferative activity against MCF-7 cancer cells, while hybrid N2, which contains a *para* zinc chelating hydroxamate moiety, could exert potent anti-inflammatory activity (NF-*κ*B inhibition, IC_50_ = 47.2 nM).

Due to the complex structure of rapamycin, CuAAC appears to be the best strategy for the generation of novel rapamycin derivatives. For example, C42 rapamycin-triazole N4 (IC_50_ = 6.05–25.88 *μ*M) could exert more potent inhibitory against H1299, MGC-803, H460, and Caski cancer cells than that of rapamycin (IC_50_ = 18.74–35.13 *μ*M) (Xie et al., 2016). Mechanism studies indicated that N4 could cause the change of cell morphological, and induce apoptosis of the tested Caski cells. Moreover, it could inhibit the mTOR signaling by downregulating mTOR phosphorylation and its downstream key proteins, P70S6K1 and S6. Thus, **N4** may have the potential to serve as a new mTOR inhibitor. C28 rapamycin-triazole **N5** (IC_50_ = 12.8–14.8 *μ*M) could also exert more potent inhibitory against A549, 769-P, ECA-109, and Caski cancer cells than rapamycin (IC_50_ = 12.3–24.5 *μ*M) (Huang Q. et al., 2018). Mechanistic studies indicated that N5 could inhibit the mTOR signaling by downregulating mTOR phosphorylation and its downstream key proteins such as P70S6K1 and 4EBP1.

Spiramycin is a natural antibiotic that is produced by *Streptomyces ambofaciens*. Spiramycin-triazole-*N*-acetylsaccharide N6 could exert potent antibacterial activity against a panel of bacterial strains (*B. subtilis, M. luteus, S. epidermidis,* and *S. pneumoniae*) with MIC values in the range of 1–4 *μ*g/ml (Klich et al., 2016). Salinomycin-triazole hybrid N7 could exert low micromolar inhibitory against Hela (IC_50_ = 0.29 *μ*M) and Caco2 (IC_50_ = 0.44 *μ*M) cancer cells (Shi et al., 2016), and the dimer N8 could exert submicromolar inhibitory activity against MCF-7 cancer cells (IC_50_ = 0.60 *μ*M) (Huang et al., 2017). Notably, both of them were more potent than that of parental salinomycin (IC_50_ = 0.32–12.99 *μ*M).

## Conclusion Remarks and Future Perspectives

As natural products are usually complex molecules with little modification space and some of them even contain labile functionalities, the structural modification of natural products with the aim to optimize their drawbacks or the construction of natural product-like drug screening libraries are the most fascinating challenges in organic synthesis. Therefore, the development of synthetic toolboxes that facilitates efficient access to the molecular diversity and unique functions of natural products is highly desirable. One such, perhaps the most successful toolbox is click chemistry, which enables the ready synthesis of a diverse set of natural product derivatives, especially the 1,2,3-triazole derivatives of terpenoids, alkaloids, steroids etc., in a highly efficient manner. Beyond the optimization of the original biological activity and the improvement of kinetics and drug-like properties, many of these derivatives have been endowed with new functions, and thereby could serve as an inexhaustible source for discoveries in drug development. In addition, click chemistry, especially the CuAAC reaction, have also been widely used in the synthesis of homodimers or heterodimers of natural products even in the presence of labile functionalities, mainly due to their high orthogonality reaction properties as compared to other chemistries such as the synthesis of amides and esters. Nevertheless, to fully utilize the power of click chemistry in natural product-based drug discovery, there remain several issues and new directions for future research in the area.

1) One of the most important merits of click chemistry is modular synthesis, which can quickly generate diverse libraries of large numbers of new compounds. However, as we can see from Figure 1, there are usually only a limited number of click chemistry derivatives that have been synthesized and screened for their functions. Thus, it would be impossible to probe the desired chemical space to generate ideal hit compounds. The reason is probably that most of the click chemistry building blocks are commercially unavailable and must be prepared. Fortunately, a 2019 paper reported a perfect solution for the synthesis of various azides by using fluorosulfuryl azide as an efficient diazo transfer reagent (Meng et al., 2019). In the future, the rational design and synthesis of modular natural product building blocks with functionalities that can react with other click chemistry building blocks in large numbers would be a useful strategy to probe the large chemical space.
2) As we can see from Figure 1, about 68% of click chemistry natural product derivatives have only been selected for anti-cancer activity, and thus their other functions are missing. In the future, it is important to be aware of the selection of the multiple functions of the natural product click chemistry derivatives against different phenotypes or targets.
3) As most of the natural product-triazole derivatives were screened by phenotypic screening (91%, Figure 1), therefore, their exact molecular targets are ambiguous. In the future, the rational design of target-based selection systems for natural product click chemistry libraries will be an important research area.
4) Beyond CuAAC click chemistry, which generates 1,2,3-triazole derivatives, some other emerging click chemistries like SuFEX chemistry have already shown their power in the generation of valuable hit molecules, and thus also could be used in natural product modification in the future.
5) Notably, another powerful hit screening technology DNA-encoded library (DEL), and especially the natural product DNA-encoded library (*n*DEL), have already shown their power in the screening of some challenge protein targets (Ma et al., 2019; Xie et al., 2020). So, if we can connect natural products with DNA-encoded libraries and diversify them by click chemistry, we could quickly generate a huge natural product derivative library with unprecedented skeleton diversity. In addition, as DEL selection is affinity-based screening, the exert molecular target of the identified hit compounds are clearly after they have been deconvoluted from the screened DEL library.
6) Because most of the natural product click chemistry derivatives were only tested in *in vitro* assays, their metabolic, pharmacodynamic, and toxicity profiles should be carefully studied in the future. For example, a recent paper reported that 1H-1,2,3-triazole containing anticancer chemotherapeutic might potentially lead to cardiotoxicity by the impairment of mitochondria (Stephenson et al., 2020).
