# Supplementary material for: Click Chemistry in Natural Product Modification
Source: Front Chem. 2021 Nov 17;9:774977. doi: 10.3389/fchem.2021.774977 (PMC8635925; doi:10.3389/fchem.2021.774977)
Supplement: Supplementary file 1 [file DataSheet1.PDF]

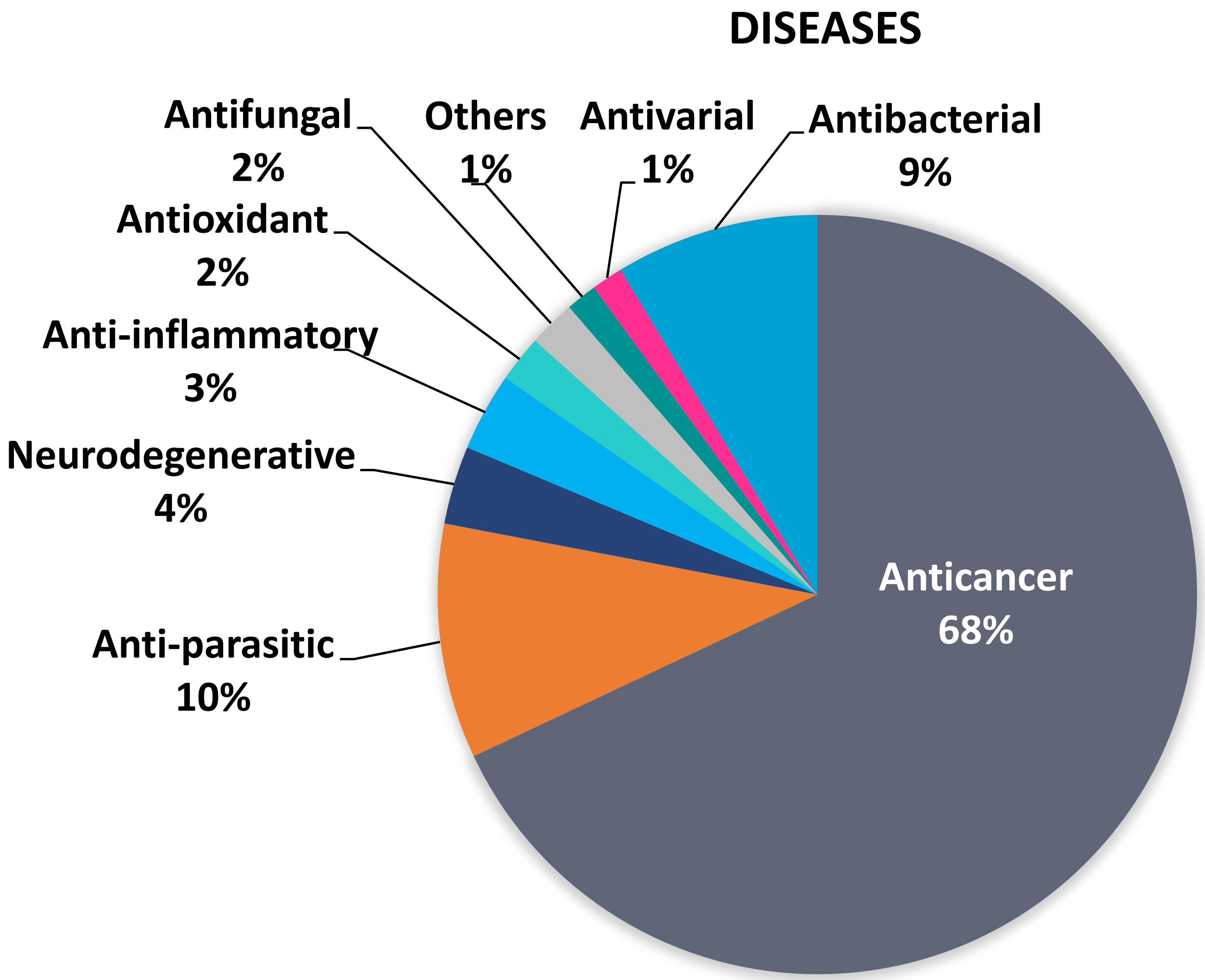

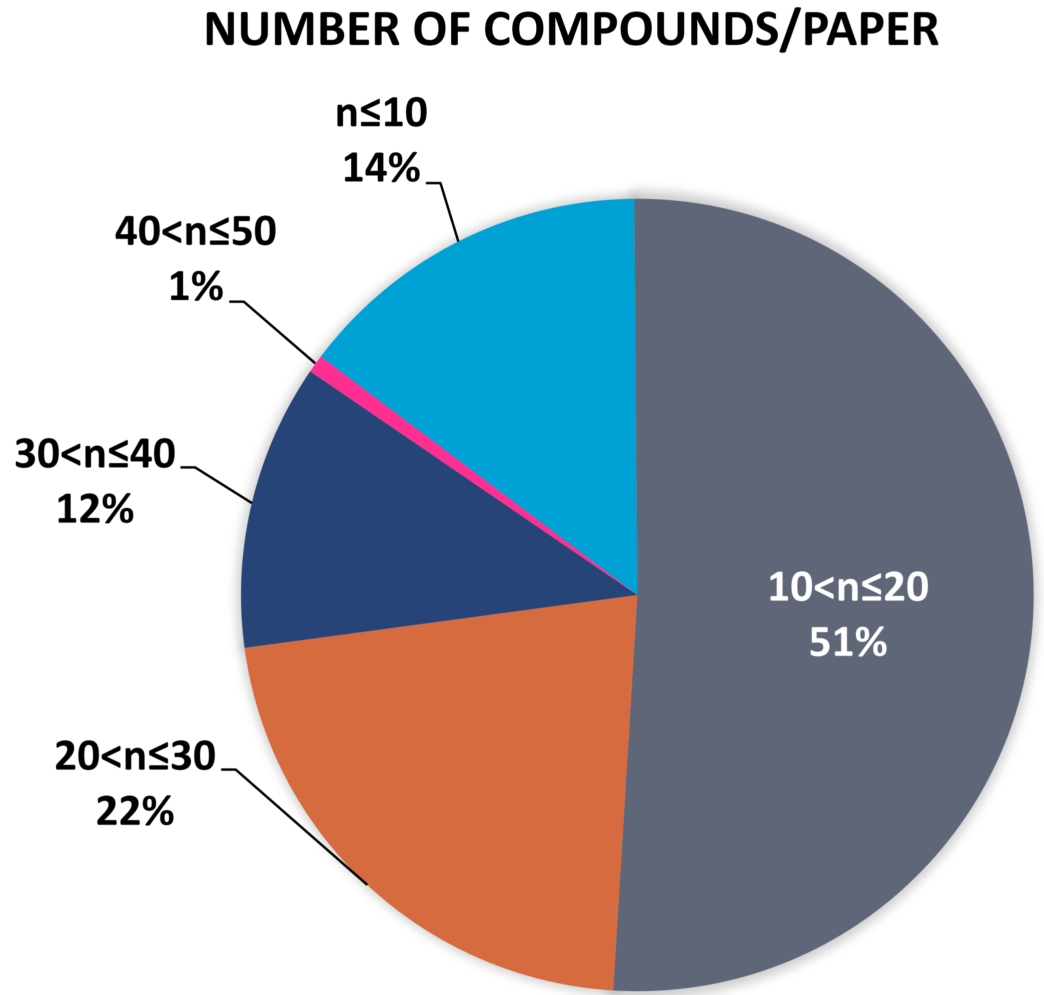

- **Click chemistry is a powerful toolbox for the modification of complex natural products**
- **Natural product click chemistry derivatives have been screened for various diseases**
